# Mechanistic read-across comes of age: a comparative appraisal of EFSA 2025 guidance, ECHA’s RAAF, and good read-across practice

**DOI:** 10.3389/ftox.2025.1690491

**Published:** 2025-12-17

**Authors:** Thomas Hartung, Costanza Rovida

**Affiliations:** 1 Center for Alternatives to Animal Testing (CAAT), Johns Hopkins University, Baltimore, MD, United States; 2 CAAT-Europe, University of Konstanz, Konstanz, Germany; 3 Johns Hopkins University, Doerenkamp-Zbinden Chair for Evidence-based Toxicology, Baltimore, MD, United States; 4 CAATevents gGmbH, Solingen, Germany; 5 TEAM mastery S.r.l., Como, Italy

**Keywords:** read-across, good read-across practice (GRAP), EFSA 2025 guidance, ECHA RAAF, new approach methods (NAM), adverse outcome pathways (AOPs), weight-of-evidence, mechanistic similarity

## Abstract

Read-across has matured from an expert-driven extrapolation based largely on structural analogy into a rigorously documented, mechanistically informed cornerstone of next-generation risk assessment. Three pivotal frameworks are compared that now shape its regulatory use: the European Food Safety Authority’s (EFSA) 2025 guidance for food and feed safety, the European Chemicals Agency’s (ECHA) Read-Across Assessment Framework (RAAF) for industrial chemicals under REACH, and the community-driven Good Read-Across Practice (GRAP) principles. Using five analytical lenses—conceptual structure, scientific rigor, implementation tools, regulatory acceptance, and practical impact—we identified areas of complementarity and divergence. EFSA provides a seven-step, uncertainty-anchored workflow that actively embeds new approach methodologies (NAMs) and adverse outcome pathway reasoning, offering applicants a transparent “how-to” template. RAAF, in contrast, operates as an evaluator’s rubric: six scenario types and associated assessment elements delineate what evidence must be delivered, thereby standardizing regulatory scrutiny but leaving dossier construction to the registrant. GRAP supplies the conceptual glue, emphasizing mechanistic plausibility, exhaustive analogue selection, explicit uncertainty characterization, and the strategic use of NAMs; its influence is evident in both EFSA’s and ECHA’s evolving expectations. (Terminology note: the acronym “NAM” was popularized at an ECHA workshop in 2016; earlier documents such as RAAF and initial GRAP papers therefore may not use the term explicitly). Regulatory experience under REACH demonstrates that dossier quality and acceptance rates rise markedly when RAAF criteria are met, while EFSA’s new guidance is poised to catalyze similar gains in food and feed assessments. Globally, the convergence of these frameworks—reinforced by OECD initiatives and NAM-enhanced case studies—signals an emerging international consensus on what constitutes defensible read-across. In conclusion, harmonizing EFSA’s procedural roadmap with RAAF’s evaluative rigor and GRAP’s best-practice ethos can mainstream reliable, animal-saving read-across across regulatory domains, paving the way for fully mechanistic, AI-enabled chemical safety assessment.

## 1 Introduction

Read-across can be implemented via two distinct but complementary modes: analogue and category approaches. In the analogue approach, a single well-justified source substance is used to fill a data gap for a structurally and mechanistically similar target. In the category approach, multiple related substances are grouped *a priori* and consistent trends (or shared mechanistic features) across the group are used to interpolate or extrapolate the target’s property. Regulatory frameworks considered here accommodate both, but differ in the degree to which they prescribe dossier construction (EFSA), evaluation (RAAF), or best-practice principles (GRAP/OECD GD 194). The read across approach is in principle applied in the same way for both toxicology and ecotoxicology assessment, even though this analysis is mainly focusing to human toxicology in the EU context.

In the evolving landscape of regulatory toxicology, read-across has emerged as a cornerstone strategy for reducing reliance on animal testing while maintaining scientific and regulatory integrity. At its core, read-across allows toxicologists to infer the properties of a chemical substance (the “target”) by leveraging known data from one or more similar substances (the “source”). This can be applied via an analogue approach, in which data from a single well-characterized source chemical are extrapolated to a target, or a category approach, in which multiple related chemicals are considered as a group to identify consistent trends for interpolation of the target’s properties.

The underlying premise—that structural and mechanistic similarity can justify extrapolation of hazard information—has long been practiced informally, but in recent years it has been codified through formal frameworks. These include first the OECD guidance from 2014, revised 2017^1^ (OECD GD 194), and the European Chemicals Agency’s Read-Across Assessment Framework (RAAF) (ECHA, 2017), developed to guide chemical safety assessments under the REACH regulation (Regulation EC 1907/2006 concerning the Registration, Evaluation, Authorisation and Restriction of Chemicals); the European Food Safety Authority (EFSA)’s 2025 guidance on read-across for food and feed safety evaluation (EFSA Scientific Committee, 2025); and the Good Read-Across Practice (GRAP) (Ball et al., 2016) principles, which emerged from academic-industry collaborations to address scientific challenges and improve methodological consistency.

Alongside Bennekou et al. (2025) and ECHA’s RAAF, this analysis is benchmarked against OECD’s Guidance on Grouping of Chemicals (OECD GD 194, 2017)—the international baseline for grouping chemicals via either analogue or category approaches. OECD GD 194 defines grouping as considering more than one chemical at a time to fill data gaps, operationalized as an analogue approach (predicting a target from one or more similar sources) or a category approach (inferring across a set showing similar or trend-based properties). It also codifies the principal data-gap filling tools—read-across, trend analysis and (Q)SAR—and provides stepwise procedures and reporting formats for documenting analogue/category justifications. Importantly, OECD GD 194 includes specific guidance for complex unknown or variable composition, complex reaction products or of biological materials (UVCBs) and how to build defensible categories for them. Therefore OECD GD 194 is used as a common denominator when comparing EFSA’s procedural roadmap, RAAF’s evaluator rubric, and the GRAP best-practice ethos.

Despite their shared goal of promoting scientifically credible read-across, these frameworks differ in structure, emphasis, and regulatory context. The RAAF focuses on standardizing the evaluation of read-across justifications, providing assessors of the REACH registration dossiers, with a scenario-based checklist of critical elements. In contrast, EFSA’s guidance aims to structure the development of a read-across argument for feed and food risk assessment, embedding it within a transparent, stepwise weight-of-evidence framework that explicitly incorporates uncertainty analysis and encourages the integration of New Approach Methodologies (NAMs). Notably, the acronym “NAM” was introduced only in 2016 at an ECHA workshop, after RAAF and the initial GRAP guidance were already published. GRAP, by contrast, is not linked to a definitive scope and operates as a conceptual and practical guide, distilling insights from case studies and regulatory experiences to recommend best practices—particularly for incorporating mechanistic reasoning, biological similarity, and toxicokinetic considerations. These differences reflect the distinct regulatory mandates of the respective bodies: EFSA’s focus on direct human exposure via food and feed necessitates more conservative decision thresholds, while REACH applications may place more weight on grouping substances for hazard classification. Understanding how these frameworks align or diverge is essential for practitioners navigating different regulatory landscapes and for harmonizing scientific approaches across sectors.

This article presents a comparative analysis of the Bennekou et al. (2025) guidance, ECHA’s RAAF, and GRAP principles, with the goal of identifying synergies, divergences, and opportunities for integration. Each framework is examined through five analytical lenses: (i) conceptual structure—how each defines and organizes the read-across process; (ii) scientific rigor—how key criteria such as structural and biological similarity, ADME properties, and mechanistic plausibility are addressed; (iii) implementation aspects—including use of case studies, templates, and supporting tools; (iv) regulatory acceptance and impact, and (v) practical utility—namely, whether the frameworks enable or hinder the routine use of read-across in practice. In doing so, the authors also reflect on the development and dissemination of GRAP, to which the authors have directly contributed, and assess its role in influencing or complementing formal regulatory frameworks. This comparison focuses primarily on EU requirements, as both documents are issued by an EU agency and an EU authority, respectively. The aim is not to exclude other approaches, but to provide a direct comparison of the documents in the context of the EU principle of “one substance, one assessment,” which applies to substances that may fall under the scope of both agencies. While the focus is on EU frameworks, the underlying principles have broader applicability, and the considerations outlined are relevant in other regulatory contexts. In this regard, OECD GD 194 provides a complementary perspective. As formally endorsed by the OECD, the guidance is expected to be accepted by all member countries, provided that the use of read-across is permitted under local regulations. Even with this limitation, the approach described in OECD GD 194 represents a wide international harmonization efforts, reinforcing the principles highlighted in the EU context.

This comparison is particularly timely in light of recent advances in toxicological science. The past decade has seen rapid progress in mechanistic toxicology, bioinformatics, and systems-level approaches, including the rise of NAMs, adverse outcome pathways (AOPs), multi-omics, and AI-enhanced predictive models such as Generalized Read-Across (GenRA) and quantitative RASAR. These advances offer powerful new tools to substantiate read-across hypotheses but also raise new questions about how frameworks should evolve to accommodate them. Understanding how EFSA, ECHA, and GRAP respond to these scientific developments is crucial for ensuring that innovation in toxicology translates into regulatory impact. By aligning structured regulatory expectations with the flexibility and scientific depth of GRAP, the field is poised to realize a harmonized, mechanistically informed, and innovation-ready future for read-across in chemical safety assessment. Noteworthy, results from *in vivo* tests for risk assessment are still necessary in many sectors and the read-across approach represents the only possibility to waive new animal tests, making this opportunity outstanding.

## 2 Comparative conceptual frameworks for read-across

This section introduces each framework’s overarching approach and philosophy, before delving into detailed comparisons in later sections (Table 1). OECD GD 194 (2014)^1^ provides the foundational, cross-regulatory blueprint for grouping and read-across, defining two complementary modes: the analogue approach (predicting a target from one or more similar sources) and the category approach (inferring across a group that shows consistent trends or shared mechanisms). Conceptually, OECD GD 194, is hypothesis-driven: a read-across/category rationale must articulate the scientific basis for similarity (structural motifs, functional groups, Absorption, Distribution, Metabolism, and Excretion (ADME)/toxicokinetics, bioactivity/Mode of Action (MoA) or Adverse Outcome Pathway (AOP) links, and relevant physico-chemical domains), set applicability/boundaries (including subcategories), and be supported by transparent data matrices and data adequacy reviews. For data-gap filling it formalizes three tools—read-across (qualitative or quantitative), trend analysis (often a local/internal Quantitative Structure Activity Relationship (QSAR) within a category), and (Q)SAR (external models)—embedded in a weight-of-evidence narrative with explicit handling of uncertainties. The guidance supplies stepwise procedures for both analogue (identify candidates → compile/evaluate data → justify similarity → document) and category formation (develop hypothesis/definition → gather and grade data → fill gaps via read-across/trends/(Q)SAR → propose minimal testing → finalize documentation via Analysis and Research Compendium/case report form (ARF/CRF) formats). Notably, it addresses complex substances/UVCBs, requiring granular composition characterization, marker constituents, and conservative handling of unknown fractions. In this comparative frame, OECD GD 194 functions as the methodological baseline against which EFSA’s procedural roadmap, ECHA’s evaluator-centric RAAF, and GRAP’s best-practice principles can be aligned.

**TABLE 1:** Comparative summary of read-across frameworks.

Feature	EFSA guidance (Food/Feed, 2025)	ECHA RAAF (REACH 2015, 2017)	Good read-across practice (GRAP, 2016)	OECD GD 194 (2007, 2017)
Framework Type	Step-by-step procedural guide (6 steps; unified for analogue or category cases)	Scenario-based evaluator tool (6 scenarios with Assessment Elements) used for category building and justification in registrations	Conceptual and flexible best-practice model	Stepwise international guidance document (covers both analogue and category grouping approaches)
Key Components	Problem formulation; target characterization; source (analogue) selection; data gap filling; explicit uncertainty analysis	Identification of analogues vs. categories; demonstration of structural and biological similarity; justification for grouping; critical review of uncertainties; assessment criteria and quantification	Emphasis on mechanistic (biological) similarity, comprehensive weight-of-evidence, transparency, and strategic use of NAMs/AOPs	Definition of category or analogue hypothesis; careful composition characterization of each substance; data collection and matrix; evaluation of data adequacy; data gap filling via read-across or testing (including QSAR support); thorough documentation of the justification
NAM Integration	NAMs actively encouraged and prioritized (use *in vitro*/*in silico* before new animal tests)	Not explicitly integrated (in 2017 RAAF), but compatible with NAM evidence if scientifically justified	Strong encouragement to integrate *in vitro* and *in silico* data (omics, AOPs, etc.) to bolster mechanistic plausibility	Implicit use of alternative data (QSAR models, analogues) is discussed; encourages use of SAR/QSAR and other non-animal data as supporting evidence
Uncertainty	Dedicated step for uncertainty characterization and predefined acceptability thresholds	Evaluated indirectly within each assessment element (uncertainties must be addressed per element)	Central focus on uncertainty: advocates explicit characterization and discussion of uncertainties and assumptions	Addressed qualitatively via weight-of-evidence – emphasizes scientific justification for predictions but no separate uncertainty quantification step in framework
Intended Users	Applicants (dossier submitters) and EFSA risk assessors; domain-specific (food/feed additives, contaminants)	Primarily regulatory evaluators (ECHA staff), though industry uses it as a *de facto* checklist for dossier preparation; industry primarily uses the RAAF for category building and justification in registrations	Broadly applicable: researchers, industry scientists, and regulators across sectors (general best practices)	Regulators and industry scientists globally; applicable across international programs (e.g., OECD High Production Volume program, REACH) for hazard assessment and data gap filling
Regulatory Status	Official guidance document (EFSA Scientific Committee)	Internal evaluator’s tool (ECHA), publicly available to guide submissions and evaluations	Non-binding consensus principles (publication in peer-reviewed literature) – influential via scientific acceptance	Official OECD guidance document – not legally binding but internationally recognized best practice
Evolution	New in 2025, incorporating EU-ToxRisk findings and updated uncertainty analysis guidance	First introduced 2015 (updated 2017); may require future revision to fully integrate modern NAM data and approaches	Originated ∼2016; refined through subsequent workshops and case study publications (e.g., Rovida et al., 2020)	First edition in 2007; expanded second edition in 2017; serves as a foundation for later OECD guidance (e.g., grouping approaches for nanomaterials) and ongoing global harmonization efforts

### 2.1 EFSA’s 2025 read-across guidance (food/feed safety)

**FIGURE 1:**
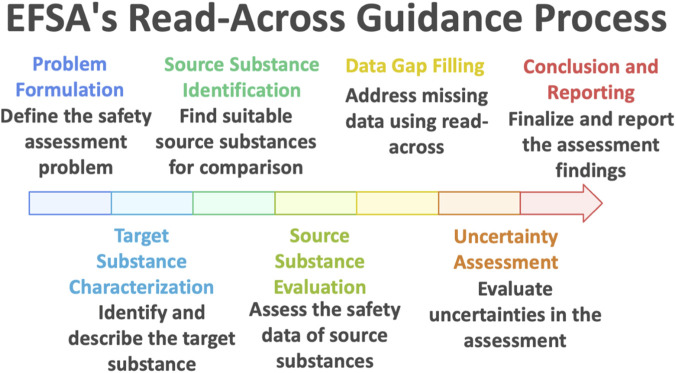
Read across process according to EFSA (2025).

Bennekou et al. (2025) guidance marks a significant step forward in formalizing the use of read-across for food and feed safety assessment. Unlike earlier, more general references to read-across in regulatory practice, this guidance provides a detailed, procedural framework intended to be used both by applicants preparing dossiers and by risk assessors reviewing them. Structured as a seven-step process (Figure 1), the framework guides users from initial problem formulation through to reporting and decision-making. Each phase—(1) Problem formulation, (2) Target substance characterization, (3) Source substance identification, (4) Source substance evaluation, (5) Data gap filling, (6) Uncertainty assessment, and (7) Conclusion and reporting—is tightly embedded within a weight-of-evidence philosophy. By doing so, the guidance ensures that all relevant information is considered and synthesized in a transparent, logical sequence, aligning with EFSA’s general principles for scientific assessment.

Conceptually, EFSA’s framework places strong emphasis on transparency, traceability, and scientific neutrality. The process begins with a careful definition of the regulatory context and problem formulation, including the specific decision question, the critical toxicological endpoint(s), and the acceptable level of uncertainty for the risk assessment at hand. This sets the stage for a clearly articulated read-across hypothesis, which must be iteratively evaluated and, if necessary, refined through the subsequent steps. Rather than treating read-across as a binary yes/no decision, the guidance views it as a hypothesis-driven method that must be continuously validated as more evidence—chemical, biological, or mechanistic—is gathered. This iterative and hypothesis-based logic distinguishes the EFSA approach from simpler structural analogies and is critical for building scientific credibility in the prediction.

A distinguishing feature of the 2025 EFSA guidance is its systematic integration of new approach methodologies (NAMs), including *in vitro*, *in silico*, and mechanistic data sources. The framework actively encourages applicants to use NAM data to strengthen or clarify key aspects of the read-across hypothesis, such as toxicodynamic similarity, metabolic convergence, or tissue distribution. Indeed, the guidance explicitly recommends exploring the applicability of NAMs as a first option before resorting to additional *in vivo* testing. This proactive positioning of NAMs reflects EFSA’s broader commitment to the 3Rs principle and aligns with emerging trends in mechanistically informed toxicology. Notably, the guidance acknowledges that while NAMs can reduce uncertainty, their use must be accompanied by appropriate validation and contextualization. To support this, the guidance encourages the incorporation of AOP-based reasoning, kinetic modeling (e.g., Physiologically based pharmacokinetic, (PBPK)), and other lines of evidence that can demonstrate biological relevance and mechanistic plausibility.

The overarching objective of the EFSA framework is to foster a read-across process that is both scientifically robust and practically applicable within the constraints of regulatory decision-making. By providing a clear procedural map, by requiring systematic evaluation of uncertainty, and by explicitly welcoming modern toxicological tools, EFSA aims to increase both the scientific defensibility and regulatory acceptance of read-across. In doing so, the guidance not only strengthens the methodological integrity of read-across within the food and feed sectors but also sets a benchmark for how sector-specific frameworks can incorporate evolving scientific standards without compromising regulatory clarity. As a result, EFSA’s framework functions not merely as a regulatory checklist but as a scientific tool for guiding sound judgment in the face of incomplete data—exactly the condition under which read-across is most often needed.

### 2.2 ECHA’s Read-Across Assessment Framework (RAAF)

**FIGURE 2:**
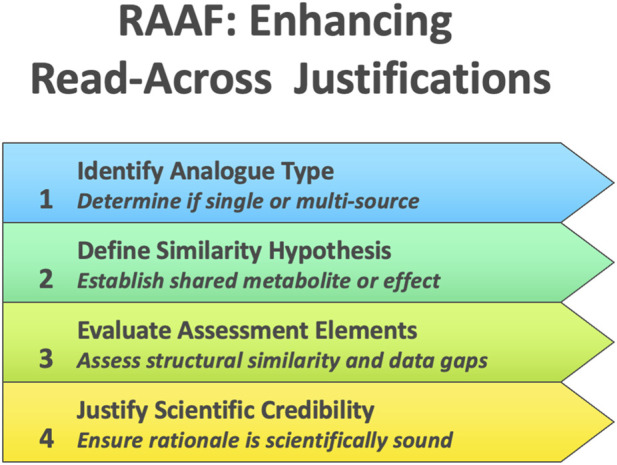
ECHA’s read-across assessment framework (RAAF).

ECHA’s RAAF represents a complementary approach to EFSA’s guidance, functioning not as a how-to manual but as a structured evaluative rubric for read-across under REACH. While not originally intended for submitters, the RAAF was from the outset recognized as shaping how companies structured their read-across arguments, as documented in the 2012 ECHA–Cefic workshop proceedings^2^. The RAAF codifies common read-across scenarios, each defined by the nature of the analogues (single-source “analogue” vs. multi-source “category”) and the hypothesis underpinning similarity (e.g., shared metabolite vs. common toxicological effect). For each scenario, a set of predefined “assessment elements” (AEs) enumerates the critical scientific considerations that an assessor must scrutinize, such as the adequacy of structural similarity, toxicokinetic comparability, and coverage of data gaps. By guiding experts to judge each element of a read-across case systematically, the RAAF ensures that the fundamental scientific principles behind the read-across are transparently evaluated in a consistent manner (Figure 2). A 2018 meeting report by Chesnut et al. (2018) highlighted regulators’ recognition of read-across as the most frequently used alternative to animal testing, while simultaneously emphasizing key obstacles to its broader use—including lack of guidance, uncertainty communication, and reproducibility concerns that were only partially addressed pre-2025. Crucially, the framework focuses on the scientific credibility of the read-across rationale itself–it delineates *what* aspects need to be justified, without prescribing *how* a practitioner should construct the read-across argument. This high-level structure was initially developed for internal use by ECHA to harmonize dossier evaluations, but it was later published to inform registrants of the “rules of the game,” thereby prompting improvements in the quality of read-across justifications submitted under REACH. The intended regulatory impact of the RAAF has been to raise the bar for acceptability by clearly identifying the evidentiary elements a valid read-across must contain. In practice, the introduction of RAAF has translated to more predictable and rigorous scrutiny of read-across cases: if a dossier’s read-across hypothesis is implausible or key supporting evidence is missing for an AE, ECHA will deem the justification inadequate, often leading to requests for additional (*in vivo*) data. By making the evaluation criteria explicit, the RAAF has thus driven both regulators and industry toward a more scientifically robust read-across process, even as it acknowledges the need for expert judgment and case-by-case flexibility in interpreting its framework.

ECHA accepted 49% of submitted read-across hypotheses for chemical toxicity during the testing proposal phase between 2008 and August 2023 (Roe, 2025). The acceptance rate is notably higher for group-based read-across approaches (about 62%) compared to analogue read-across types (about 39%).

Key factors influencing acceptance^*2*^ include the structural similarity between target and source substances—read-across proposals with very high similarity had acceptance rates over 75%, while those with lower similarity dropped to about 31%. Acceptance rates were also initially higher (2012–2015) but declined after ECHA’s Read-Across Assessment Framework (RAAF) first publication in 2015 and updated in 2017 as the process became more standardized.

### 2.3 Good read-across practice (GRAP)

**FIGURE 3:**
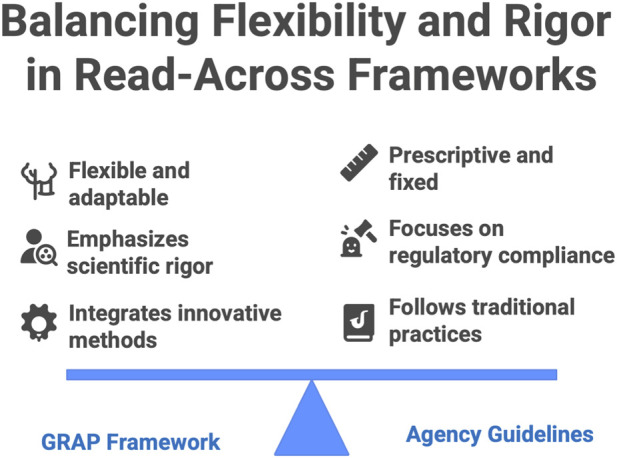
Good read-across practice aiming to balance flexibility and rigor.

Parallel to these agency-led frameworks, the concept of Good Read-Across Practice (GRAP) emerged from an academic–industry collaboration as a consensus-driven best practice guide for developing read-across arguments. Introduced in 2016 by Ball and colleagues (Ball et al., 2016), GRAP was conceived as a flexible yet rigorous framework to maximize the scientific robustness and transparency of read-across, complementing official guidelines. Instead of a fixed procedural template, GRAP provides overarching principles and a conceptual structure that can be tailored to different regulatory contexts while upholding high standards of evidence. A central tenet is the emphasis on scientific plausibility: a GRAP-compliant read-across must demonstrate a sound rationale for expecting similar hazard outcomes between source and target chemicals, which in turn hinges on a well-substantiated basis for similarity (structural, biological, or both). To this end, the GRAP approach encourages exhaustive and impartial exploration of potential source analogues and data. All plausible source substances should be considered, and any exclusion or inclusion of data must be transparently justified, thereby avoiding cherry-picking of evidence. This comprehensive weight-of-evidence strategy is coupled with an insistence on explicit uncertainty analysis: at each stage, and especially in the final read-across prediction, residual uncertainties should be clearly characterized and quantified where possible. By documenting every step–from initial analog selection criteria to the rationale for rejecting or accepting certain data points–GRAP seeks to make the reasoning process as reproducible and unbiased as possible (Figure 3).

In terms of technical innovation, GRAP brought attention to integrating NAMs (e.g., high-throughput *in vitro* assays, computational predictions) into read-across as supportive evidence (Zhu et al., 2016), foreshadowing later regulatory trends toward NAMs. Indeed, the GRAP guidance highlighted how mechanistic bioactivity data and modern cheminformatics tools can bolster the justification for similarity between substances. This forward-looking incorporation of novel data streams, along with its formal strategies for uncertainty communication, marked a step beyond earlier broad guidance by providing a more nuanced template for what a “scientifically rigorous” read-across should entail. Although not a regulatory mandate, GRAP has had a notable influence on the community’s expectations. Its publication and subsequent international workshops (Chesnut et al., 2018; Rovida et al., 2020) underscored that adhering to these best-practice principles could significantly improve the odds of regulatory acceptance of read-across cases. Regulators have indirectly welcomed GRAP insofar as it addresses many shortcomings previously seen in read-across submissions (for example, opaque reasoning or insufficient justification of similarity) by advocating greater clarity, completeness, and methodological discipline. The conceptual flexibility of GRAP means it can be applied across different jurisdictions and chemical domains, serving as a unifying reference that aligns industry practice with the overarching scientific standards valued by bodies like ECHA and EFSA. In summary, GRAP functions as an academic and industry-derived conceptual framework that, while less prescriptive than agency guidelines, reinforces and extends the core principles necessary for credible read-across, ultimately aiming to bridge the gap between innovative hazard prediction approaches and their regulatory trustworthiness.

UVCBs pose specific challenges for read-across due to compositional variability. Recent guidance converges on three pillars: (i) granular composition characterization of both source and target, ideally identifying all ≥1% constituents and bounding the unknown fraction; (ii) a mechanistic hypothesis or category rationale linking constituents or shared structural features to the predicted hazard; and (iii) transparent uncertainty treatment when constituent levels differ or compositional variability is non-negligible. ECHA’s 2022 advice on UVCB read-across highlights that similarity may be established where mixtures share identical constituents in different proportions or distinct, structurally analogous constituents—provided differences do not affect the endpoint. OECD GD 194 (2014) likewise emphasises defining category boundaries (e.g., carbon range, distillation range), identifying marker constituents, and using data matrices, local QSARs or potency-weighted constituent information to support predictions.

### 2.4 Read-across for UVCB substances (complex mixtures)

Regulatory read-across frameworks have traditionally dealt with defined single- or multi-constituent chemicals, but UVCBs pose unique challenges. UVCBs are characterized by complex, partially undefined compositions and inherent variability, making it difficult to establish the “sufficient similarity” required for read-across. Recent guidance initiatives–notably a 2022 ECHA advisory document on read-across for UVCBs and the OECD’s 2014 chemical grouping guidance–provide direction for applying read-across principles to these complex substances.

ECHA’s 2022 guidance (following a 2021 REACH Annex revision) lays out stringent conditions for UVCB read-across. It emphasizes that structural similarity must be demonstrated on the level of individual constituents. In practice, this means all major constituents of the UVCB need to be identified and compared across substances. The guidance specifies that every constituent present at ≥1% w/w should be identified, and the identified constituents must collectively account for at least 80% of the UVCB’s mass. If more than ∼20% of a UVCB’s composition is unknown or uncharacterized, “it is not possible to establish structural similarity” for the purpose of read-across. Two modes of comparing UVCBs are recognized: in some cases the source and target UVCBs may share identical constituents in different proportions; in others, they may have different constituents that are nevertheless structurally analogous to each other. Both situations require demonstrating that any differences in composition (or in constituent concentrations) do not materially affect the property being predicted. By definition, UVCBs exhibit composition variability; however, the guidance insists that this variability must be bounded and understood. Constituent concentrations should be measured in multiple samples to characterize variability, and differences in the levels of specific constituents between source and target must be factored into the similarity argument. Simply showing that two UVCBs contain a common list of constituents is insufficient–one must also show comparable levels or a rationale why differing concentrations (or unknown fractions) do not alter the hazard in question. Importantly, if a known hazardous constituent is present, its concentration across the category should be carefully considered (and often matched or treated as worst-case) in the read-across justification. These provisions underscore that read-across for UVCBs demands a much more granular understanding of composition than for traditional single-component substances.

OECD GD 194 grouping guidance similarly addresses complex substances by outlining best practices for forming a “category” of analogues when chemicals are multi constituent substances or UVCBs. A key initial step is to clearly define the category and its members, which for UVCBs often means identifying a set of representative single-constituent analogues that span the range of components present in the complex mixtures. The guidance stresses detailed composition characterization to the extent practicable–for example, specifying critical compositional parameters such as carbon-chain length ranges, boiling point or distillation ranges, or other descriptors that delineate category members. Any known or generic description of the mixture’s makeup should be provided, and marker chemicals (signature constituents) should be identified and quantified in each member of the group if possible. This helps ensure that each complex substance in the category can be directly compared via common reference points. The OECD framework also advises identifying representative constituent compounds covering the diversity of structures present, and flagging any constituents with outlying properties (for instance, a particular component known to drive a specific toxicity). Such outliers may need to be addressed with additional data or conservative assumptions. When it comes to filling data gaps for category members, the OECD notes that read-across or trend analysis can often be applied qualitatively within a well-defined category, whereas fully quantitative predictions may be more uncertain for UVCBs. In some cases a “local QSAR” or toxic equivalence factor approach can be developed, using data on individual constituents to predict multi constituent behavior. For example, if each complex substance can be represented as a combination of certain constituent profiles, one might employ potency estimates of those constituents (or surrogate compounds) to estimate the mixture’s overall hazard. The guidance acknowledges that quantitative read-across for UVCBs is challenging, but it is feasible to establish at least a bounding range of hazard values or to use a weighted approach if mechanistic understanding allows. Throughout, transparent documentation is emphasized: the category justification for UVCBs should clearly explain how structural similarity is assessed (e.g., what compositional parameters are considered “similar enough”), how any missing information is extrapolated, and what uncertainties remain due to the unknown fraction or variability of composition.

In summary, both the ECHA 2022 advice and OECD 2014 guidance converge on certain key principles for UVCB read-across. They require a rigorous characterization of composition (to minimize the “unknown” portion and ensure comparability), insist on a mechanistic hypothesis or rationale linking the category members (why their shared constituents or structural features would yield similar toxicological profiles), and provide for careful treatment of uncertainties arising from variable or unidentified constituents. While applying read-across to UVCBs is inevitably more complex than for defined single chemicals, these guidances offer a pathway to do so scientifically. They encourage the use of all available analytical data, computational tools, and theoretical considerations to build a case that even complex, variable substances can be grouped and assessed as a category. With these additional safeguards and explanations in place, regulators can gain confidence in read-across predictions for UVCBs, enabling hazard and risk assessments to proceed with reduced animal testing even for substances that challenge our traditional notions of chemical identity.

Under the European Union’s REACH Regulation, the assessment of UVCBs relies heavily on a clear and consistent definition of substance identity and on the use of structured read-across or category approaches, as articulated in the ECHA RAAF (ECHA, 2017). REACH requires that similarity among UVCBs be scientifically justified, typically through well-characterized composition data, shared manufacturing processes, or demonstrated common mechanisms of toxicity. By contrast, under the U.S. Toxic Substances Control Act (TSCA), the Environmental Protection Agency (EPA) has historically addressed UVCBs—through the experience gained in the category-based evaluations under the *High Production Volume (HPV) Challenge Program* (Stanton and Kruszewski, 2016). These U.S. approaches emphasize pragmatic grouping of substances with comparable source materials and processing histories, sometimes with less reliance on precise compositional characterization than under REACH. Consequently, while both systems aim to facilitate data gap filling and reduce unnecessary testing, their regulatory acceptance criteria, evidentiary expectations, and practical implementation of read-across for UVCBs differ markedly, reflecting divergent regulatory philosophies—one emphasizing formalized justification and documentation (REACH), and the other prioritizing category pragmatism and precedent-based decision-making (TSCA).

EFSA’s read-across guidance was not considered because it explicitly excludes UVCBs from its scope, with their assessment deferred to future revisions. Nevertheless, the guidance notes that read-across may be acceptable when the manufacturing process is highly similar, as is the case for petroleum distillates. It also acknowledges the OECD guideline, which includes UVCBs within its scope.

## 3 Scientific rigor in read-across: criteria and considerations

This section evaluates how each framework addresses the core scientific issues that determine read-across credibility. Subsections focus on key themes (biological similarity, ADME, uncertainty, NAM/AOP integration), comparing EFSA, ECHA/RAAF, and GRAP side by side.

### 3.1 Establishing biological similarity and mechanistic plausibility

EFSA: The guidance defines structural and mechanistic similarity as the cornerstone for selecting source analogues. In practice, during *Step 2 (Target characterization)* and *Step 3 (Source identification)*, EFSA expects assessors to gather all relevant data on the target’s physicochemical properties, toxicodynamic behavior (mode of action, key target organs, etc.), and toxicokinetics. This information shapes a hypothesis for similarity: e.g., “Target and source share a structural motif that is responsible for toxicity” or “Target metabolizes to the same reactive intermediate as Source, leading to the same adverse outcome.” EFSA’s framework encourages an iterative refinement–if during Step 4 (Source evaluation) a mechanistic dissimilarity emerges (say the source has an effect via a pathway not relevant to target), one might refine the hypothesis or select a different source. The guidance explicitly mentions the use of AOPs as a way to frame the similarity rationale. The overarching idea is that EFSA does not treat read-across as a simple structural analogy exercise; it must be biologically plausible that the substances will cause the same effects in the same way.

ECHA (RAAF): The RAAF’s assessment elements put heavy weight on mechanistic plausibility. For any scenario, one of the first elements assessed is typically: “*Is the hypothesis of why the substances are expected to have similar properties explained and justified?*” This means a registrant must clearly state the basis of the read-across–often a combination of structural similarity and some evidence (or logical argument) of similar mode of action or metabolism. For example, RAAF Scenario 1 (analogue approach) requires checking if the source and target share the functional group or metabolite responsible for the toxicity in question. If a structural difference exists (e.g., one analog has an extra ring or different substituent), RAAF expects an explanation of why that difference is not expected to affect toxicity–possibly by pointing to an AOP or mechanistic understanding. RAAF also distinguishes between toxicity that is explained by a known mechanism versus cases where it’s empirical but consistent; in the former, the read-across is stronger if you can articulate the mechanism (e.g., both chemicals are pro-estrogens that metabolize into an active estrogenic compound). In summary, RAAF operationalizes mechanistic similarity as a checklist item–a read-across without a mechanistic justification is likely to be deemed less robust or even unacceptable. This insistence has pushed registrants to include more biological reasoning, aligning with the GRAP philosophy.

GRAP: From the outset, GRAP has championed a shift “*from structural similarity alone to biological similarity*”. Zhu et al. (2016) introduced the concept of using broad biological data (e.g., high-throughput screening profiles, toxicogenomics, cell-based assays) to establish common activity between source and target chemicals. A term that arose from these efforts is “bioactivity-based read-across” (sometimes abbreviated as *BaBRA*), meaning one uses *in vitro* bioactivity signatures to group chemicals or identify analogues with similar biological profiles. This is essentially leveraging NAM data to bolster the mechanistic plausibility of read-across. GRAP publications encourage constructing an argument that includes known or hypothesized modes of action and even linking those to formal AOPs if available. For instance, if two chemicals trigger similar pathways *in vitro* (say both activate oxidative stress responses in cells), that can support a hypothesis that they might cause the same *in vivo* outcome (if that pathway is known to lead to toxicity). Additionally, in the 2020 workshop (Rovida et al., 2020), experts emphasized that demonstrating absence of unexpected “biological activity” is important if one wants to be confident in a negative prediction; this means casting a wide net for mechanistic similarity, including checking multiple endpoints or using *omics* to ensure no hidden differences. In practical terms, GRAP encourages assembling a mechanistic similarity weight-of-evidence: e.g., similar *in vitro* assay responses, similar metabolic pathways (enzyme interaction data), and any relevant *in vivo* info (like both cause liver toxicity in the same pattern). This multi-faceted approach to mechanistic similarity is now reflected in EFSA’s guidance (which explicitly gathers toxico-dynamic and -kinetic info) and is something RAAF implicitly rewards (a strong mechanistic case will satisfy multiple assessment elements).

### 3.2 Toxicokinetic and ADME considerations

EFSA: The inclusion of toxicokinetic data (ADME) is explicitly mentioned in EFSA’s workflow. In *Step 2 (Target characterization)*, assessors are advised to compile what’s known about the target’s kinetic behavior, and similarly for sources in *Step 4*. EFSA highlights that differences in ADME between source and target can critically undermine a read-across if not accounted for. For example, if the source compound is poorly absorbed and therefore not toxic, but the target is well absorbed, one cannot read-across the “non-toxic” result unless additional justification is given (perhaps the target is also expected to be low in bioavailability due to some property, or the exposure scenario differs). EFSA’s guidance likely suggests conducting *in vitro* metabolism studies or using PBK (physiologically based kinetic) modeling as needed to compare kinetic profiles. The iterative nature of EFSA’s process means if a discrepancy in metabolism is found (say, target generates a toxic metabolite that source does not), the approach might shift–maybe exclude that source or generate more data. In short, EFSA requires proactive evaluation of ADME similarity as part of the hypothesis: the target and source should have comparably bioavailable forms at the target site of action, or differences must be quantitatively accounted for in the uncertainty analysis.

ECHA (RAAF): ADME is a recurring theme in the RAAF assessment elements. One specific assessment element (present in several scenarios) asks: “*Have differences in metabolism or toxicokinetics between source and target been addressed?*” The assessor will look for statements like “Both substances are metabolized via the same pathway,” or “The target has no structural features that would lead to different reactivity or bioactivation than the source.” If a registrant ignores a known kinetic difference (e.g., one is a pro-drug that needs metabolic activation and the other is not), RAAF would flag that as a major flaw. The RAAF guidance (2017) itself was written when NAM-supported ADME data were less commonly provided, but it does incorporate traditional knowledge–for instance, if source and target have significantly different molecular weight or lipophilicity, has the impact on absorption been discussed? If one is rapidly excreted and the other accumulates, how does that affect the prediction? In essence, RAAF enforces that a credible read-across must compare toxicokinetic profiles. Cases exist where ECHA accepted read-across because applicants convincingly argued kinetic similarity or adjusted their hypothesis to account for kinetic differences (one example, as reported by Ball et al., 2016, was a category of substances where bioavailability differences were used to select worst-case testing members–showing regulators do pay attention to these details).

GRAP: The GRAP initiatives identified “*ADME differences as one of the most challenging issues in read-across”*. In their analysis, even if two chemicals are structurally similar, differences in absorption or metabolism can lead to different toxicity outcomes, which has been a reason some read-across arguments fail. Therefore, GRAP strongly advises practitioners to deeply investigate ADME: use available tools (like *in silico* predictors for metabolism, *in vitro* hepatic clearance assays, etc.) to see if the target might behave differently inside the body than the source. If differences are found, GRAP suggests either refining the category (maybe the outlier should not be used as a source) or incorporating the difference into the prediction (for example, applying an uncertainty factor if the target might have higher internal exposure). The 2020 workshop report explicitly notes that NAMs can help with ADME–e.g., performing *in vitro* metabolic stability or transporter assays to compare substances. It also hints at future use of PBPK modeling fed by NAM data to quantitatively handle kinetic differences. Another GRAP concept relevant here is “toxicokinetic applicability domain” – ensuring the target lies within the domain of sources in terms of kinetics (Patlewicz et al., 2014, described the idea of local validity, which in reverse can be applied to say: if all sources share a kinetic trait, ensure target does too, or otherwise justify). Moreover, when arguing *lack* of toxicity via read-across, GRAP experts insist on an even more detailed ADME discussion, basically “cover all bases” to show no unaccounted toxic metabolites will appear. Summing up: GRAP pushes for comprehensive ADME evaluation, a practice that EFSA and ECHA frameworks are increasingly incorporating, thereby aligning with GRAP on this front.

### 3.3 Treatment of uncertainty and weight-of-evidence

EFSA: Uncertainty analysis is a distinct step (Step 6) in EFSA’s read-across workflow, underscoring how central it is. By the time one reaches this step, the guidance expects that all sources of uncertainty from previous steps have been identified and, where possible, quantified or characterized. EFSA explicitly asks whether the *overall uncertainty can be reduced to tolerable levels* for the decision at hand by using established approaches (e.g., applying assessment factors) or by generating additional data such as NAM outputs. In practical terms, EFSA assessors will look at uncertainties like: differences between source and target (residual questions about mechanism or kinetics), quality of the data used (is the source study reliable or does it introduce uncertainty?), and relevance of the endpoint extrapolation (e.g., read-across from subchronic to chronic toxicity might add uncertainty). The EFSA guidance likely provides a framework or at least discussion on how to document these uncertainties and perhaps an uncertainty table or narrative (see EFSA Scientific Committee general guidance on uncertainty analysis in risk assessment^3^). Another important aspect: EFSA’s step 1 (Problem formulation) ties into uncertainty by setting an acceptable level of uncertainty at the outset–for instance, a screening assessment might tolerate more uncertainty than a definitive risk assessment for regulatory approval. This contextual approach to uncertainty is something relatively unique in EFSA’s guidance (tailoring the rigor to the context). Finally, EFSA concludes with either accepting the read-across (if uncertainties are within acceptable bounds) or recommending filling the data gap with actual testing if uncertainties remain too high. The emphasis is on *transparent communication* of uncertainty so decision-makers know how much confidence to place in the read-across conclusion.

ECHA (RAAF): While RAAF does not have a separate “uncertainty” step, uncertainty is woven throughout its assessment elements. Each missing justification or unaddressed difference is essentially an uncertainty. The RAAF document uses language like “*it is unclear whether …* ” to signal uncertainties that would make the read-across hypothesis weaker. One might say RAAF’s grading of each element (fully addressed vs. not) is a way of qualifying uncertainty: if something is not addressed, the uncertainty in that aspect is unacceptably high. ECHA’s broader guidance on read-across (e.g., Chapter R.6 of the IR&CSA Guidance) also talks about describing uncertainties and using a weight-of-evidence approach. Weight-of-Evidence (WoE) is often how uncertainties are mitigated: if multiple independent pieces of evidence support the prediction, the uncertainty is effectively reduced. Ball et al. (2016) noted that ECHA tends to be more comfortable when read-across is part of a WoE with other data. Indeed, ECHA’s official stance (as also mirrored in OECD guidelines) is that read-across is stronger when combined with other non-test data–for example, if you have two analogues showing the same effect and also a QSAR model predicting the effect, plus perhaps some *in vitro* test indicating similar activity, then the total WoE is compelling even if each piece alone has uncertainty. In terms of regulatory acceptance, ECHA may accept a read-across argument with some uncertainty if the consequence of being wrong is still protective (e.g., using read-across to confirm a hazard exists might be accepted with less stringent proof than using read-across to assert safety). The EFSA guidance actually cites that point: a weaker justification might suffice to confirm hazard (since regulators default to conservative stance), but a much stronger justification is needed to conclude “no hazard” via read-across. This philosophy is shared by ECHA: declaring something non-toxic by read-across requires high confidence (low uncertainty), whereas identifying a potential hazard by read-across is more readily acceptable, since it errs on the side of caution. In summary, RAAF does not provide a quantitative uncertainty analysis framework, but by ensuring all critical questions are answered, it implicitly demands uncertainties be addressed. If not fully addressed, the case is likely rejected or requires additional data (which is effectively saying the uncertainty was too high).

GRAP: Handling uncertainty is one of GRAP’s central themes. The guidance and related papers emphasize that uncertainty should be explicitly addressed in read-across justifications. This includes acknowledging assumptions (e.g., “we assume impurity profiles of the source and target do not significantly influence toxicity–a source of uncertainty”), data variability, and knowledge gaps. GRAP recommends tools such as Klimisch scores for data reliability (Schneider et al., 2009) and describing confidence in each line of evidence. It also promotes constructing weight-of-evidence (WoE) narratives or scoring systems to balance evidence, approaches further developed in later work (e.g., EFSA Scientific Committee, 2025; R.I.V.M. uncertainty toolbox).

The 2020 Rovida workshop expands on this by proposing a categorical system (low/medium/high) to rate uncertainty across domains and recognizes RAAF’s role in highlighting these elements. Rovida et al. describe this as a “pragmatic solution,” noting that ECHA’s RAAF already captures many of these aspects, even if not labeled as formal uncertainty analysis.

Transparency is another GRAP priority: uncertainties can be acceptable if clearly communicated and the overall approach remains conservative. GRAP also advises supplementing uncertain elements with additional evidence—e.g., *in vitro* metabolism or mechanistic assays. By proactively addressing uncertainties, submitters strengthen their read-across cases. Overall, GRAP aligns closely with EFSA’s formal uncertainty step and complements RAAF by encouraging applicants to anticipate and mitigate issues assessors would likely flag.

### 3.4 Incorporation of NAMs (new approach methodologies) and AOP frameworks

EFSA: The 2025 EFSA guidance is one of the first regulatory frameworks to integrate NAM usage into the read-across process. It advocates for exploring new approach methods at multiple junctures: e.g., *prior to animal testing for data gap filling* (the guidance explicitly notes “*new data refer to exploring NAMs first before considering any in vivo testing*”, and for reducing uncertainty in various steps. EFSA’s Appendix likely provides examples of NAM integration–for instance, using *in vitro* bioassays to confirm that target and source elicit similar biological responses, or using computational models (QSARs, read-across tools like the OECD QSAR Toolbox^4^ (Kuseva et al., 2019) to identify potential analogues and predict properties. The guidance’s emphasis on “standardised approaches” includes the use of internationally recognized NAMs where available (e.g., OECD Test Guidelines for *in vitro* tests, validated QSAR models) to support read-across. Another modern aspect is incorporation of AOP thinking (Leist et al., 2017): by aligning the read-across hypothesis with an AOP, assessors can see that upstream key events are shared between source and target. EFSA, being a scientific risk assessor, is receptive to AOP frameworks–for example, if a read-across argument is that two pesticides cause liver toxicity through a common AOP involving PPAR-α activation, and one can show NAM evidence of both activating that pathway *in vitro*, that strengthens the case. In summary, EFSA’s approach not only allows but encourages NAM and AOP use to make read-across more mechanistically anchored and to avoid unnecessary animal tests. This reflects the contemporary shift in toxicology towards Next-Generation Risk Assessment (NGRA) paradigms, and EFSA is signaling that its door is open to such data.

ECHA (RAAF): At the time of RAAF’s development (2015 and 2017), NAMs were not explicitly integrated. RAAF is method-agnostic and it focuses on the quality of the justification, regardless of whether the evidence is from traditional tests or new methods. However, since RAAF is used by expert assessors, they can certainly consider NAM evidence as fulfilling certain assessment elements. For example, one assessment element might be “*Is there evidence that the source and target have a similar toxicological profile?*“. In classic cases this might be answered with both having, say, a positive in the same *in vivo* assay. But NAM data like ToxCast^5^ bioactivity fingerprints could also be used as evidence of similar profiles (if properly rationalized). ECHA has separately been involved in initiatives to incorporate NAMs (such as defining conditions under which *in vitro* or *in silico* can suffice), but these are often captured in guidance documents outside the RAAF (like the 2018 guidance on using *in vitro*/*ex vivo* for skin sensitization, etc.). It’s worth noting that ECHA and EFSA have collaborated on some NAM fronts (for example, EFSA’s mention of integrating NAMs likely draws from experiences in REACH and research projects like EU-ToxRisk^6^ (Escher et al., 2019; 2022), which involved ECHA. The RAAF’s structure does not exclude the use of NAMs. When NAM-derived data are used to fill an endpoint for a source substance, the RAAF can still be applied to assess the plausibility of the read-across; however, the registrant must demonstrate that the NAM result is valid and relevant. Noteworthy, RAAF assessors require that NAM data be explicitly linked to the toxicological endpoint being addressed and to the underlying similarity hypothesis. Ongoing discussions (as of 2025) aim to update the RAAF to better accommodate NAM-supported read-across. Regulatory uptake of NAMs remains gradual, but frameworks such as EFSA’s may help accelerate integration by providing practical examples of how mechanistic and *in vitro* data can be formally incorporated.

GRAP: The GRAP publications and workshops have been strong proponents of NAMs to enhance read-across. Even in 2016, GRAP discussions included how high-throughput screening data (like those from ToxCast) could identify mechanistic similarities or outliers in categories. Zhu et al. (2016) essentially provided a toolkit of NAM types (*in silico* predictions, in chemico assays, cell-based assays, -omics technologies) and how each could support various aspects of read-across. Fast-forward to Rovida et al. (2020): they explicitly talk about “NAM-enhanced RAx” as the next evolution. The NAM-enhanced read-across workflow from EU-ToxRisk (featured in that paper) is a concrete outcome of GRAP thinking–it formalizes adding NAM data generation into the process (e.g., after initial analog identification, perform targeted *in vitro* tests to confirm similarity or probe a hypothesis). GRAP sees NAMs as not just nice-to-have, but often *essential* for certain read-across cases, especially where traditional data are missing or when trying to demonstrate a negative (no effect) with confidence. For instance, if you want to read-across that a new food additive has no genotoxicity based on an analogue, GRAP would encourage conducting some *in vitro* genotoxicity screens or mechanistic assays in both compounds to bolster that argument, rather than relying solely on analogue’s *in vivo* result. Also, GRAP encourages the use of computational NAMs: read-across can be complemented by QSAR predictions (ensuring the target is within the applicability domain of the model) or by docking studies if relevant (say both source and target binding to the same receptor, demonstrated via modeling). In terms of AOPs, GRAP and associated efforts have frequently linked read-across case studies to AOPs to illustrate mechanistic reasoning. The concept of mechanistic applicability domain ties directly to AOPs–essentially ensuring that the chemical group defined makes sense in light of known biological pathways. By doing so, one can argue the read-across has a sound scientific foundation rather than being a blind correlation.

Summary Alignment: All three approaches are increasingly convergent here: EFSA explicitly instructs the use of NAMs/AOPs, ECHA (via RAAF) implicitly accepts them if they strengthen the case, and GRAP has been advocating for them from the start. This alignment is a positive trend–indicating a collective move in toxicology toward modern, mechanism-based safety assessment. Rovida et al. (2020) see NAMs as “*the most important innovations to improve acceptability of RAx*”, reflecting the consensus in the community that this is the way forward. Confirmation arrived also from a meeting organized by the EU-ToxRisk project, which convened representatives from regulatory institutions and representatives of the industry to discuss how NAMs can be applied to improve acceptability of read-across, by providing experimental justification of similarity (Rovida et al., 2021).

## 4 Practical implementation and guidance usability

This section compares how each framework handles the “on-the-ground” aspects of applying read-across. It covers the availability of case studies and examples, any tools or templates provided to users, the level of detail and user-friendliness of each guidance, and overall clarity.

### 4.1 Use of case studies and examples

EFSA: The 2025 EFSA guidance includes or is accompanied by examples to help users understand application. Within the guidance document itself, EFSA might use illustrative examples in appendices or figures–e.g., a figure showing possible situations where read-across can be applied in EFSA’s remit (like regulated products vs. contaminants) and a figure outlining the stepwise process with an example flow. These examples serve to clarify abstract concepts (like what a good source substance evaluation looks like, or how to report uncertainty). The presence of case studies indicates EFSA’s intent to make the guidance practical and didactic, not just theoretical. It’s likely that EFSA drew on cross-sector experience (REACH cases, pesticide assessments, etc.) to shape scenarios relevant to food safety (for example, reading across toxicity from a well-studied flavor chemical to a newly proposed flavor with similar structure). By providing examples, EFSA lowers the barrier for applicants to attempt read-across, because they can model their approach on successful templates.

ECHA (RAAF): In the original RAAF documentation, ECHA provided generic scenario descriptions and included simplified case illustrations for each scenario type. Additionally, since RAAF’s release, various supporting materials have been developed. For example, the OECD QSAR Toolbox^4^ (a tool widely used for read-across) has built-in guidance for identifying the applicable RAAF scenario and prompts related to those assessment elements. There are also webinars and published case studies demonstrating how to construct a read-across that passes RAAF scrutiny. In literature, retrospective analyses of REACH dossiers (e.g., “A Systematic Analysis of Read-Across Adaptations … ” or specific case analysis papers) effectively act as case studies highlighting why certain read-across proposals succeeded or failed. So while ECHA’s RAAF document itself might not be as tutorial in style as EFSA’s guidance, the community around it (including ECHA’s own publications and third-party guidance) has generated many examples. Notably, ECHA sometimes publishes illustrative decisions or reports (anonymized) – these show, for example, an instance of Scenario four category that was accepted, and what the key reasoning was. The article can mention that industry also contributed to case studies: e.g., ECETOC’s case study reports^7^, or the OECD’s series of read-across case studies^8^, many of which involve European regulators. These case studies often reference RAAF in evaluating the outcome. Thus, for a practitioner, resources exist to see how a case similar to theirs was handled. The RAAF framework’s strength is that if you know your situation fits, say, Scenario 3, you can consult published examples of Scenario three read-across to guide your own.

GRAP: The GRAP publications were rich in examples by design. Ball et al. (2016) reviewed ECHA’s decisions on actual dossiers to draw lessons–effectively summarizing dozens of cases in terms of what went right or wrong. Zhu et al. (2016) likely presented example uses of biological data to support read-across (perhaps showing how a toxicity pathway was confirmed in both source and target by *in vitro* tests). Moreover, GRAP was not just papers–it included two workshops (Chesnut et al., 2018; Rovida et al., 2020) where participants, including regulators, discussed case studies in depth. The 2020 Rovida workshop also collated case study insights on an international scale (bringing in examples from different regulatory regimes like cosmetics, pharma, etc.). Additionally, GRAP thinking has been demonstrated in projects like EU-ToxRisk, which published case study papers (some referenced in Rovida et al., 2020) showcasing NAM-supported read-across in practice (Rovida et al., 2021). All this to say, practical demonstration has been a key part of GRAP–the concept is grounded in learning from real examples. By comparing, EFSA’s foray into read-across is recent and will benefit from these existing examples, whereas GRAP compiled them to push the field forward. Hopefully, EFSA continues to gather and publish case studies as the guidance is implemented, similar to how REACH has built a knowledge base of cases over time.

### 4.2 Tools and templates for implementation

EFSA: The 2025 EFSA guidance provides a structured, seven-step framework for conducting and documenting read-across in food and feed safety assessment. These steps—ranging from problem formulation to conclusion and reporting—are intended to be followed in sequence and serve as the backbone of a well-documented justification. EFSA expects that each step be addressed in the final report, effectively functioning as a *de facto* template for applicants. For instance, Step 1 (Problem Formulation) calls for defining the regulatory context, hazard endpoint, and acceptable uncertainty; Step 2 (Target Characterization) requires compiling all relevant identity, physicochemical, and toxicity data for the target substance; and Step 3 (Analogue Identification) involves presenting the criteria and process for selecting appropriate source substances. This structured format promotes transparency, consistency, and scientific traceability.

To support systematic documentation, the guidance encourages the use of tabular tools such as data matrices to compare target and source substances and highlight data gaps. It also includes a dedicated template for uncertainty assessment (Appendix C), based on EFSA’s broader uncertainty guidance, allowing users to categorize uncertainties and evaluate their impact on the read-across conclusion. While EFSA does not provide a stand-alone “read-across report template” or IT interface, it explicitly recommends the use of established tools—such as the OECD QSAR Toolbox—for identifying analogues and compiling supporting data. These tools help facilitate reproducible analogue selection and provide structured formats for data analysis.

The guidance does not contain sector-specific reporting templates for individual regulatory domains (e.g., food additives or pesticides), but it is intended to be broadly applicable across food and feed safety areas. Applicants are expected to integrate the read-across framework into their respective submission formats. Overall, EFSA’s guidance contributes significantly to harmonizing and strengthening the read-across process by providing a clear procedural roadmap, emphasizing comprehensive documentation, and aligning the scientific justification with established principles of uncertainty analysis and weight-of-evidence reasoning.

ECHA: ECHA’s primary contribution to read-across practice has been the RAAF document, which functions as a rigorous evaluation framework rather than a fill-in template. The RAAF (first introduced in 2015 and updated in 2017) defines six scenario-specific approaches (two analogue-based and four category-based) and outlines the critical scientific elements that a valid read-across justification must address. These elements cover key aspects like establishing chemical and mechanistic similarity between source and target, articulating a clear read-across hypothesis, demonstrating data adequacy, and accounting for uncertainties. Notably, RAAF provides high-level principles and a checklist of questions for assessors, but it does not prescribe a reporting format or step-by-step template for registrants. In practice, companies registering chemicals under REACH have translated RAAF’s requirements into their own internal templates. A typical REACH read-across justification document will mirror RAAF’s evaluation criteria and ensure that all necessary evidence is presented for ECHA’s review. ECHA’s IUCLID software, used for dossier submissions, explicitly includes fields for read-across justification in each endpoint record. Registrants must identify analogues and provide a scientific rationale in these fields, guided by ECHA’s instructions (e.g., describing analogue identity, toxicological similarity, etc.). Indeed, ECHA’s technical completeness check will flag a dossier if an endpoint is waived by read-across without an adequate justification text or identified source substances, underscoring that such documentation is mandatory. Tools have also evolved to support the assembly of read-across justifications: for example, the OECD QSAR Toolbox incorporates the RAAF logic, prompting users to select the appropriate RAAF scenario and listing the information needed for that scenario. In summary, while EFSA is now proposing a brand-new formal template, ECHA’s framework comes with a decade of hard-won experience. This has yielded many “informal” templates and digital workflows aimed at satisfying RAAF’s requirements and achieving regulatory acceptance. One persistent gap has been the absence of a publicly available standard template issued by regulators; nonetheless, the strong alignment between EFSA’s stepwise template and ECHA’s RAAF criteria hints at a future convergence, where a common read-across report format might serve both agencies’ need.

GRAP: The GRAP initiative provides a set of guiding principles for crafting scientifically robust read-across justifications. Unlike EFSA’s or ECHA’s formal submission frameworks, GRAP is not tied to a specific dossier format; instead, it outlines the components that any well-founded read-across argument should contain. Key recommendations from GRAP closely parallel the expectations of regulators. For instance, a read-across justification should begin with a clear statement of the read-across hypothesis (the toxicological rationale linking source and target substances) and thoroughly document the analogue selection process. All potential source chemicals should be considered and the reasons for including or excluding each should be transparently reported to avoid any perception of “cherry-picking” data. GRAP emphasizes comprehensive data gathering for each analogue, covering the spectrum of relevant endpoints–especially critical if one is trying to demonstrate an absence of hazard. Any differences or inconsistencies in data between source and target must be openly discussed. In fact, an analysis of ECHA dossier failures showed that the most common reasons for read-across rejections were exactly issues that GRAP seeks to prevent insufficient supporting evidence, poorly justified similarity arguments, and inadequate substance identity characterization. To address such pitfalls, GRAP guidelines enumerate fundamental elements that should underpin every read-across justification: unambiguous identification of both target and source substances, a mechanistic explanation linking chemical structure to the predicted effect, a robust and unbiased dataset supporting the hazard prediction, and an explicit accounting of uncertainties. These tenets map closely to the criteria in ECHA’s RAAF and the content of EFSA’s template, meaning that following GRAP’s advice inherently moves a practitioner toward fulfilling both agencies’ requirements.

Over the years, experts involved in GRAP have also proposed more structured ways to document read-across, moving toward a standardized “read-across report” format. For example, some have suggested creating a formal Read-Across Assessment Report analogous to a QSAR Prediction Reporting Format (QPRF), with predefined sections to ensure completeness and reproducibility of the rationale. The GRAP workshops and subsequent publications introduced tools to improve clarity and transparency, such as visualizing chemical similarity relationships (e.g., using dendrograms or heatmaps of bioactivity) and tabulating side-by-side comparisons of source and target data, so that regulators can easily inspect trends and gaps in the read-across argument. Although GRAP itself did not issue an official template document, it effectively laid the blueprint that both ECHA and EFSA have built upon. In line with GRAP’s concepts, the EU-ToxRisk research program has been developing a next-generation “RAx” guidance aiming to integrate NAMs into read-across justification. This forthcoming guidance–worked out by a consortium of industry, academic, and regulatory experts–is expected to recommend a harmonized reporting structure for read-across that accommodates novel data streams (*in vitro* assays, -omics, etc.) alongside conventional toxicity data. Indeed, GRAP highlighted the value of structured reporting and even called for publicly available templates and example cases to help practitioners achieve regulatory compliance. A logical recommendation arising from these efforts is the creation of a unified template that merges the strengths of all three approaches: EFSA’s stepwise narrative, ECHA’s RAAF-based checkpoints, and GRAP’s emphasis on comprehensive evidence and clear reasoning. In practice, a scientist who follows the GRAP guidance when assembling a read-across case will likely satisfy the content requirements of EFSA’s template and address ECHA’s RAAF evaluation criteria by default. The convergence of GRAP’s best practices with regulatory frameworks ultimately improves the credibility and acceptance of read-across, steering the field toward a more standardized and transparent future.

### 4.3 Level of detail and clarity of guidance

EFSA: The Bennekou et al. (2025) guidance is notable for its clarity and completeness, spanning more than 60 pages and systematically detailing each of the seven steps in the read-across workflow. It offers comprehensive explanations of key concepts—such as problem formulation, toxicokinetic and dynamic similarity, and the tolerability of uncertainty—and provides structured expectations for documentation at each stage. By embedding complex topics such as NAM integration and uncertainty quantification within a sequential framework, EFSA ensures that even relatively unfamiliar readers can follow the process step by step. This pedagogical design reflects EFSA’s recognition of its broad stakeholder audience, which includes applicants from the food and feed sectors who may have limited experience with read-across methodologies. The guidance frequently reiterates principles of scientific transparency and impartiality, encouraging applicants to present information in a manner that is not only robust but also independently reproducible. Although the level of detail may appear daunting to some, the document’s structure—augmented by narrative explanations, tabulated summaries, and an appended uncertainty assessment template—supports user navigation and practical application. The clarity of the guidance benefited from a prior round of public consultation and an expert stakeholder workshop in early 2025, which helped refine its terminology and address interpretative ambiguities. In sum, EFSA has achieved a rare balance of procedural precision and scientific nuance, creating a document that is both instructive and operationally relevant.

ECHA (RAAF): In contrast, the ECHA RAAF provides a shorter but more technical framework focused primarily on evaluation. It is composed of a core ∼30-page document and supporting appendices, organized around six scenario types and their associated Assessment Elements (AEs). Each scenario outlines the structure of the read-across to be assessed (e.g., one-to-one analogue vs. category-based prediction), and each AE poses specific questions that an evaluator must answer—for instance, whether the similarity rationale is clearly established or whether uncertainties are adequately addressed. This format offers clarity to experienced evaluators but may appear opaque to newcomers, particularly as it provides minimal guidance on how to actually construct the read-across argument. The RAAF assumes familiarity with underlying toxicological concepts and does not define terms like “common mode of action” or “toxicokinetic similarity,” which may limit accessibility for less experienced users. Supplementary documents, including illustrative examples and explanatory notes, have gradually improved usability, as has the broader context provided by ECHA’s IR&CSA Chapter R.6 guidance^9^. Nevertheless, users must often triangulate multiple documents to obtain a full understanding of read-across expectations under REACH. This fragmented structure contrasts with EFSA’s consolidated format, making RAAF potentially more challenging to navigate despite its conceptual precision. Still, its logical design and scenario-driven rigor are widely appreciated by practitioners familiar with the REACH regime, and its alignment with formal IUCLID fields and regulatory workflows lends practical coherence to the read-across assessment process.

GRAP: The GRAP publications, beginning with Ball et al. (2016) and Zhu et al. (2016), offer a different type of clarity—one based not on regulatory prescription, but on conceptual guidance and scientific rationale. These documents, published in narrative style aim to educate rather than mandate. They define the major challenges in constructing defensible read-across cases—such as inadequately justified analogue selection, failure to consider biological similarity, or unacknowledged uncertainty—and then propose solutions grounded in best scientific practices. GRAP does not prescribe a specific reporting format but provides detailed examples, tables, and checklists that can serve as *de facto* templates for practitioners. For instance, Ball et al. outline essential components such as a clearly articulated hypothesis, systematic analogue identification, robust weight-of-evidence construction, and transparent communication of assumptions and uncertainties. The clarity of GRAP lies in its accessibility: it uses plain scientific language, abundant references, and illustrative case discussions to make complex issues understandable, even for those new to regulatory read-across. While it lacks the procedural authority of EFSA or ECHA documents, it fills a critical pedagogical role by explaining *why* each element matters and *how* it can be addressed using evolving tools and data types—including NAMs, AOPs, and *in silico* approaches. This makes GRAP an indispensable resource for improving scientific literacy and reasoning among both applicants and assessors.

Taken together, the three frameworks differ in their orientation but are complementary in practice. EFSA’s guidance tells applicants *what to do* and *how to report it*; ECHA’s RAAF explains *what regulators will check* and *why it matters*; and GRAP articulates *why the underlying science must be rigorous and transparent*. By combining EFSA’s procedural roadmap with RAAF’s evaluation logic and GRAP’s conceptual insights, practitioners can assemble scientifically credible and regulatorily compliant read-across arguments. The growing convergence among these sources, particularly around principles of biological similarity, transparency, and uncertainty analysis, suggests an opportunity for future harmonization of guidance that supports both scientific innovation and regulatory clarity.

## 5 Regulatory acceptance and impact

Having discussed frameworks and their scientific/practical aspects, the regulatory impact and acceptance of read-across under these frameworks is examined next, and whether they facilitate the wider use of read-across. This section contrasts how read-across is received and utilized under the regulatory regimes of REACH (chemicals) and EFSA (food/feed), and how GRAP has influenced or been integrated into regulatory thinking. It will also touch on global regulatory implications where relevant.

### 5.1 Acceptance and impact in EFSA’s domain (food/feed risk assessment)

Historically, EFSA has been more cautious in its use of read-across for food and feed chemical risk assessments. In the absence of formal guidance until recently, read-across was used only sparingly and under tightly controlled conditions. One notable context was the evaluation of flavoring substances, where regulators have for decades applied an analogue approach–grouping flavor chemicals by structural similarity and inferring safety of one from another–to fill data gaps^10^. Even so, such practices were largely confined to flavorings and certain well-understood groups, and EFSA’s scientific panels generally preferred direct experimental data for decision-making on food additives, feed additives, and other substances that directly affect human or animal health. In areas like pesticides, for example, read-across was virtually never accepted for an active substance’s hazard assessment; only in specific cases like the safety evaluation of metabolic by-products (e.g., grouping pesticide metabolites to assess genotoxicity) did EFSA explicitly allow read-across in its conclusions. This conservative stance stemmed from the high bar for certainty in food/feed safety–without clear guidelines, panel experts were reluctant to rely on analogies due to the potential risks of uncharacterized uncertainty in consumer protection contexts.

The landscape began to change with the development of Bennekou et al. (2025) guidance on read-across, which signals a more formal acceptance of the approach in food and feed risk assessments and outlines a clear workflow. It provides concrete tools such as lists of applicable *in vitro* methods, templates for analyzing and documenting uncertainties, and example case studies to illustrate successful read-across justifications. By formalizing these elements, the 2025 guidance aims to enhance the scientific robustness and transparency of read-across evaluations, aligning with the EU’s commitment to the 3Rs. Notably, EFSA’s guidance builds on principles similar to ECHA’s RAAF and OECD frameworks–emphasizing that structural similarity must be backed by biological plausibility and thorough documentation (analogous to the RAAF’s criteria). The very existence of this guidance is significant: it indicates EFSA’s institutional endorsement of read-across as an acceptable component of weight-of-evidence assessments, whereas previously panels had no formal benchmark for such approaches.

The true test will be how EFSA’s panels and working groups apply the guidance. Because EFSA’s remit is broad (from regulated products where an applicant is motivated to get approval, to contaminants where data may be sparse and no one “owns” the problem), the acceptance might vary by context. The guidance acknowledges that the acceptability of read-across depends on context and problem formulation. For example, EFSA doing a risk assessment on a contaminant may have no choice but to use read-across if that’s the only data source (making it a necessary option), whereas in a food additive application, EFSA could ask the applicant to do a test unless a convincing read-across is presented. The guidance itself likely will make EFSA assessors more receptive, because it gives them a clear checklist akin to RAAF to evaluate submissions. So in the short term, EFSA is expected to pilot the acceptance of read-across in upcoming evaluations (maybe even retrospective application to some pending dossiers). Indeed, EFSA working groups have already begun deliberating how to apply read-across in challenging contexts–for instance, considering analogues for complex mixtures like food enzymes, which vary in production strain and composition. These discussions, and the guidance’s insistence on case-by-case evaluation with strong justification, suggest that applicants who follow the 2025 guidance closely may find a greater willingness by EFSA to accept read-across *in lieu* of certain studies. The authors expect to see read-across used more formally in areas such as feed additives and novel food ingredients, especially when a proposed substance has well-known analogues with data. In the flavorings sector, what was once an informal read-across practice is now backed by official guidance, likely increasing regulators’ comfort in relying on those analogies. Overall, EFSA’s attitude appears to be shifting from one of general skepticism to a cautiously positive stance: read-across can be an integral part of risk assessment so long as it is performed with rigor and transparency as per the guidance. This not only harmonizes EFSA’s approach with ECHA’s to some degree, but also encourages more proactive use of non-animal data in food safety decisions (a domain where ethical and practical pressures to minimize animal testing are growing).

It is important to note the difference in regulatory consequences between REACH and EFSA’s domains, as this influences how read-across is applied and accepted. Under REACH, a failed read-across (i.e., one that ECHA does not accept as adequate evidence) typically results in a request for further testing–usually the registrant must conduct the standard *in vivo* study to fill the data gap. While this incurs cost and time burdens, the chemical can often remain on the market in the interim, meaning the primary penalty is a compliance delay rather than an immediate market removal. In EFSA’s food/feed assessments, however, an unaccepted read-across can halt an authorization entirely. If a company submits a read-across *in lieu* of a toxicity study for a new food additive or feed additive and EFSA’s panel is not convinced of its validity, the outcome may be that safety cannot be demonstrated and the product is not approved for use. In such cases EFSA would likely issue an opinion that more data are needed, effectively putting the application on hold until the applicant generates the missing information. Thus, the tolerance for uncertainty is lower: EFSA panels tend to err on the side of caution (“when in doubt, require data”) because a positive regulatory decision directly affects public health protection. The historical hesitancy by EFSA to embrace read-across can be partly explained by these stakes–unlike a REACH registrant who can test later, an EFSA-regulated entity might have no product to market if their read-across is rejected. The new 2025 guidance addresses this by delineating how to reduce uncertainty (through rigorous analysis and documentation of assumptions), hopefully enabling EFSA to approve substances with read-across-supported evidence without compromising on safety. In summary, EFSA’s formal guidance is poised to improve acceptance of read-across in its domain by clarifying the rules of the game, even as the agency’s underlying mandate–to only authorize products proven safe–ensures that the standard of proof for read-across remains appropriately high compared to REACH.

### 5.2 Acceptance and impact in REACH (industrial chemicals regulation)

REACH and ECHA (RAAF) Context: Under REACH, read-across is legally recognized in Annex XI of the regulation as a valid adaptation to standard information requirements, provided substances are similar such that their properties can be predicted from one another. The early development of the RAAF was strongly influenced by stakeholder dialogues, notably a 2012 workshop co-hosted by ECHA and Cefic LRI^2^, where industry and regulators agreed that the RAAF—even though designed as an evaluator tool—could serve to guide dossier preparation and improve justification quality. RAAF was introduced to bring rigor to the evaluation of these adaptations. Its impact was immediately felt after 2017: Many registration dossiers that had proposed read-across were re-examined or clarified under the new framework. Initially, acceptance rates of read-across were not high–analysis up to 2015 showed a lot of read-across proposals were rejected or queried by ECHA due to insufficient justification (Ball et al., 2016). RAAF, in a sense, enabled ECHA to articulate why those were insufficient (lack of mechanism, etc.) and communicate that to registrants. Over time, as industry began using RAAF as a guide, the quality of read-across justifications improved, and so did acceptance. This is evidenced by the proportion of cases in later dossier evaluations where read-across is accepted outright or with minimal questions, as opposed to earlier years. RAAF also had a side effect: some companies, seeing the high bar, chose to do more testing rather than attempt read-across, arguably a negative impact on 3Rs. However, others invested in better documentation and supplementary experiments (like targeted *in vitro* tests) to meet the bar, which is in line with the intention of REACH to promote alternative methods but only when scientifically valid.

ECHA’s evaluations (and analyses like Ball et al., 2016) found that companies often failed to explain how structural differences between source and target chemicals affect toxicity or to justify the inclusion/exclusion of substances in a category. By codifying such requirements, the RAAF directly targeted these deficiencies, thereby improving dossier quality and consistency in read-across submissions. There are clear signs that this framework has had a positive impact on acceptance rates under REACH. Early in the REACH process, acceptance of read-across adaptations was low due to the shortcomings noted above. Notably, read-across proposals based on well-defined chemical categories (grouping several analogues with consistent toxicological trends) have significantly higher odds of acceptance than one-to-one analogue approaches, even if this data derives only from the analysis of the testing proposal submitted in the REACH registration dossiers (Roe, 2025). This trend suggests that the RAAF’s emphasis on rigorous category justification and scientific plausibility is translating into greater regulatory trust when those criteria are met. The overall quality of read-across dossiers has improved, and both industry and regulators have gained confidence that, if a read-across is documented in line with RAAF principles, it can serve as a reliable alternative to new testing.

At the same time, the stringent standards of the RAAF have created a regulatory feedback loop and some unintended hesitation. ECHA scrutinizes every dossier that waives standard data via read-across, and if a read-across rationale is deemed implausible or insufficiently supported, ECHA will request additional (often *in vivo*) data from the registrant. These compliance checks, which ECHA intensified after 2019, have prompted companies to refine and bolster their read-across justifications in subsequent submissions–a dynamic that gradually elevates quality. However, the high bar for acceptance can also discourage marginal cases: some registrants choose to conduct new tests rather than risk a rejected read-across. Indeed, only about 23% of testing proposals submitted to ECHA between 2008 and 2023 even attempted an adaptation like read-across, reflecting a cautious approach by industry. This indicates that while the RAAF increased clarity, it also made read-across a resource-intensive option that companies may reserve for strong cases. On balance, ECHA’s framework has brought greater rigor, consistency, and predictability–a positive in terms of building regulatory trust–but it also means read-across must clear a substantial evidentiary hurdle. Importantly, RAAF reflects international guidelines and practices, for example, the OECD’s chemical grouping guidance (2007^11^ and 2014^12^), which incorporated similar criteria for analogue vs. category approaches and highlighted the use of mechanistic data in justifying read-across. In this way, the robust standards set under REACH via the RAAF have increased global regulatory confidence in read-across and fostered more convergence in how read-across is evaluated worldwide.

Regulatory Confidence: One measure of acceptance is how often regulators challenge read-across. Under RAAF, if a company follows it well, the outcome is more predictable–ECHA will likely accept the read-across or at least not flatly reject it. Before RAAF, decisions might have seemed more case-by-case. Now, because RAAF is internal standard operating procedure at ECHA, there’s a level of regulatory confidence and consistency. The EFSA guidance explicitly notes that acceptability of read-across predictions can vary by regulatory context, implying that what might be acceptable under REACH might differ under EFSA. For instance, REACH is often hazard-focused (classification and labeling) and risk assessment with safety factors, whereas EFSA often needs a precise risk assessment (especially if humans will be directly exposed through food). Under REACH, a read-across might be accepted to waive an animal test if the hazard can be characterized sufficiently conservatively. In terms of impact, RAAF has ensured that when read-across is accepted under REACH, it’s generally trusted–because it has gone through a stringent filter. This trust is crucial, as it feeds back into more use: companies see that if they do it right, it works, which encourages further read-across proposals.

Challenges: The RAAF is not formally a public regulation–it’s an internal tool made public for transparency. Some argue it should be more formally integrated into guidance (though it effectively is, via references). As science evolves (NAMs, AOPs), ECHA may need to update RAAF or supplement it. But as of 2025, RAAF remains the *de facto* backbone of regulatory acceptance of read-across in chemicals.

Another key challenge in implementing read-across and grouping is the difficulty and expense of obtaining source data, which in some cases can preclude their application despite a strong theoretical basis. Data access, valuation, and cost-sharing obligations under REACH are major barriers to broader uptake of category read-across.

### 5.3 Integration and influence of GRAP on regulatory acceptance

While GRAP is not a regulatory framework, it has indirectly influenced acceptance by educating and aligning the community. Ball et al. (2016) effectively laid the groundwork for GRAP by analyzing why many early REACH read-across cases failed and by proposing solutions. That analysis found the most significant factors for rejection were a lack of sufficient supporting information and poor articulation of scientific plausibility–in other words, companies often did not thoroughly justify why source and target chemicals could be expected to behave similarly, or they provided inadequate toxicological evidence to back the read-across. GRAP was formulated to address these gaps. It emphasizes meticulous documentation and rationale at each step of a read-across argument: defining the target substance’s identity and purity clearly, systematically identifying and screening potential analogues, justifying the selection of source substances (and transparently explaining any exclusions), and assessing any uncertainties or data gaps in a reproducible way. The GRAP approach also advocates using templates and structured formats for read-across reports to ensure no critical element is overlooked and to make the logic traceable for regulators. In essence, GRAP distilled a “checklist” of scientific and regulatory considerations that, if followed, greatly increase the credibility of a read-across argument. This has raised the overall standard of read-across submissions and provided a reference point for regulators when evaluating them. The earlier ECHA-Cefic-LRI workshop^2^ explicitly called out the lack of agreed methods for uncertainty characterization and called for systematic templates—needs directly addressed by the GRAP principles and Bennekou et al. (2025) uncertainty framework. The GRAP papers (Ball et al., 2016; Zhu et al., 2016) were central to stakeholder workshops summarized in Chesnut et al. (2018), which helped define both practical and conceptual hurdles that subsequent guidance frameworks have sought to overcome.

Many regulators and industry scientists involved in GRAP carried those ideas into their organizations. For example, some GRAP co-authors are from regulatory agencies (EPA, etc.) and committees. GRAP publications have been widely cited, including in regulatory guidance: the EFSA guidance references the contributions of EU-ToxRisk and implicitly GRAP, acknowledging that efforts like GRAP and EU-ToxRisk’s RAx guidance provide input on best practices. By identifying common pitfalls, GRAP helped regulators articulate what they need to see (as codified later in RAAF and EFSA’s doc).

For instance, the EU-ToxRisk project worked on an advisory read-across reporting template complementary to ECHA’s RAAF, directly reflecting GRAP principles by helping registrants provide all necessary information in a structured format. EFSA’s new 2025 guidance likewise bears the fingerprints of GRAP: it incorporates many of the same concepts, such as rigorous uncertainty analysis and mechanistic justification, which were championed in GRAP discussions and publications. Notably, EFSA and ECHA have even jointly applied read-across concepts in certain cases–a prominent example is their 2018 joint guidance on identifying endocrine disruptors in pesticides/biocides, which explicitly included read-across as an accepted line of evidence. This cross-agency guidance indicates a harmonized understanding (consistent with GRAP) that a well-substantiated read-across can be a valid component of regulatory hazard assessment. In short, GRAP has provided a unifying thought framework that regulators across domains are using to refine their own processes: ECHA’s internal assessors use GRAP-like checklists to review dossiers, and EFSA’s experts now have a GRAP-informed roadmap for considering read-across in risk assessments. The net effect is a more coherent and agreed-upon set of standards for read-across, reducing the previous ambiguity that surrounded its use.

### 5.4 Acceptance under different regimes

Read-across and grouping are applied with varying degrees of acceptance across regulatory contexts.

In the cosmetics sector, where animal testing is banned in the EU, read-across has become indispensable. GRAP has provided the rigor needed for such assessments, and its principles are now embedded in approaches. Cosmetic safety assessors routinely group ingredients (e.g., fragrance chemicals or additives) and argue by analogy, applying GRAP’s structured justification of similarity. The Scientific Committee on Consumer Safety (SCCS) has even engaged read-across experts to support evaluations—evidence of growing regulatory confidence that, with GRAP-level diligence, consumer protection can be maintained without new animal data.

In pharmaceutical regulation, acceptance is more limited due to the conservative nature of the field and the reliance on *in vivo* testing. Nonetheless, read-across principles appear in cases such as the grouping of impurities or metabolites, where conducting additional animal studies would be impractical or unethical. Regulators like EMA and FDA have signaled openness to scientifically justified alternatives, and companies increasingly apply GRAP-like approaches internally—using computational alerts and structural analogies during drug discovery to flag potential toxicities early.

In the U.S. regulatory context, GRAP’s influence is visible in the growing integration of NAMs, even if there is still no regulatory formal acceptance. A notable example is the U.S. FDA’s evaluation of dietary supplements such as kratom, where computational models were used to compare plant-derived constituents to known opioid compounds. This form of *in silico* read-across, combining existing and modeled data, exemplifies GRAP’s call to integrate multiple evidence streams for robust risk assessment.

Overall, CAAT’s GRAP concept has helped shape thinking across domains—from cosmetics to pharmaceuticals to broader U.S. regulatory practice. The clear parallels between EFSA’s 7-step framework, ECHA’s RAAF, and GRAP’s earlier recommendations illustrate a convergent evolution toward what can now be considered good read-across practice across sectors.

Global Acceptance: GRAP also aimed at international harmonization. For instance, the concept of “*validated NAM for regulatory toxicology*” in Rovida et al. (2020) speaks to getting read-across (with NAM support) recognized broadly, not just EU. So, when multiple jurisdictions start to trust read-across as a result of improved methodology, GRAP deserves some credit for that shift. For example, the Canadian regulatory approach and US EPA’s read-across practices have evolved in parallel, often citing similar best practice papers (including those from GRAP contributors like Patlewicz et al., 2014). Outside Europe and North America, formal regulatory frameworks for read-across remain limited, and practical implementation is still emerging.

Internationally, the tenets of GRAP have been taken up and promoted by key regulatory cooperation bodies, leading to broader uptake and alignment. The OECD’s guidance on chemical grouping and read-across was updated in 2017 in line with emerging best practices, many of which mirror GRAP recommendations. For example, the OECD framework stresses documenting analogue suitability and, where possible, incorporating mechanistic or biological data to support the category hypothesis–a concept emphasized by both Ball et al. and the complementary paper by Zhu et al. (2016) that explored integrating biological information into read-across. The OECD has also facilitated an international read-across case study project to build confidence and share experience among regulators in different jurisdictions. Through such projects (and under the umbrella of ICATM, the International Cooperation on Alternative Test Methods^13^), regulators from Europe, North America, and Asia have compared notes on how to apply read-across, aiming for mutual acceptance of credible read-across approaches. The US EPA, for instance, has actively contributed to and benefited from these global discussions. Likewise, agencies such as the US EPA’s Office of Pollution Prevention and Toxics, which has long used chemical categories in its New Chemicals Program, have developed analogous category-based assessment tools (e.g., the Analog Identification Methodology (AIM)^14^ and Chemical Assessment Clustering Engine (ChemACE)^15^ in line with the read-across concept. to systematically facilitate read-across evaluations. These tools and the EPA’s emerging “Generalized Read-Across” algorithm (GenRA)^16^ reflect the same GRAP ideals: using large toxicological databases and *in silico* models to strengthen the scientific underpinning of read-across predictions. Canada and other countries have similarly engaged via the OECD in adopting harmonized approaches, even if their regulations are at different stages of formally embracing read-across. The overarching result is that GRAP has helped forge international consensus on what a good read-across looks like, which in turn is slowly translating into aligned regulatory guidance and greater acceptance across the board.

In summary, GRAP has significantly shaped regulatory acceptance of read-across by driving convergence toward high standards and shared practices. It has harmonized the understanding of what makes a read-across scientifically credible and how to document it, thereby increasing the likelihood that a robust read-across argument will be accepted by different authorities. There is now an explicit international recognition that if a read-across is done to GRAP standards, it ought to be accepted broadly–it should not happen that one jurisdiction approves a read-across case that another, with no scientific disagreement, rejects arbitrarily. As one commentary put it, it would be “unacceptable that a good RAx [read-across] would be, for example, accepted in the EU and rejected in Japan or *vice versa*”, underscoring the need for global harmonization. Thanks in large part to GRAP, organizations like the OECD are taking the lead in coordinating policies so that regulators move in step towards this goal. While challenges remain and some sectors are slower to change, the influence of GRAP is evident in the increasingly aligned guidance, workshops, and cross-talk between regulators worldwide. By promoting rigorous science and transparency, GRAP has enhanced regulatory confidence in read-across as a valid alternative approach. In the long run, this thought leadership contributes to a more unified international regulatory environment–one in which a well-substantiated read-across is recognized and trusted as a legitimate means to fill data gaps, ultimately supporting innovation and the 3Rs without compromising safety.

## 6 Do these frameworks enable or hinder read-across uptake?

In this section, the existence of these structured frameworks is critically evaluate whether it is facilitating greater use of read-across or by transmitting the perception that the final goal is too difficult to reach, thereby discouraging its use. Each approach’s impact on the practical uptake of read-across is considered.

Bennekou et al. (2025) Guidance–Enabler or Barrier? On one hand, EFSA’s guidance is clearly an enabler: it gives permission and a clear method to use read-across in food safety assessment where previously assessors might have been unsure or skeptical. By formalizing the process, it legitimizes read-across as a scientific approach under EFSA’s remit. It also explicitly supports integrating new methods (which many scientists are eager to use) – that modern angle can draw more practitioners to attempt read-across, knowing EFSA is open to innovation. The structured nature (7 steps) acts as a helpful scaffold, especially for less experienced risk assessors. On the other hand, the guidance is demanding: to properly follow all steps with thorough analysis might require significant effort (data gathering, possibly generating NAM data, conducting uncertainty analysis). Smaller companies or groups with limited resources might find it challenging, potentially deterring them unless they have strong incentives. Also, if EFSA sets a very high standard for “tolerable uncertainty”, it might result in many proposed read-across cases being deemed insufficient, which could discourage future attempts. However, given EFSA’s public health focus, it’s expected they’d rather be conservative. The key point is EFSA’s guidance enables use by providing a roadmap, but whether it increases uptake will depend on how user-friendly it turns out to be in practice and how often EFSA accepts cases (early success stories will encourage others).

ECHA’s RAAF–Necessary Rigor or Discouraging Complexity? When RAAF was introduced, some in industry felt it was a hurdle because it highlighted so many potential weaknesses in a case that many existing read-across justifications fell apart under scrutiny: In that sense, RAAF initially hindered some uses of read-across–companies either had to invest more effort or abandon the read-across approach for certain endpoints (opting to test instead). Over time, however, RAAF has also been an enabler by educating industry on how to do read-across properly. It forced a learning curve that has led to higher competency in the field. Now, many companies proactively apply RAAF internally (a cultural change toward more rigorous science), which leads to better dossiers and ultimately *more* successful read-across. In effect, RAAF weeded out the sloppy usages but strengthened the credible ones. If the measure of uptake is “number of accepted read-across cases,” initially that may have dipped, but now it’s climbing as people adapt. Another aspect is that RAAF’s complexity might dissuade newcomers–for example, small enterprises without specialized toxicologists might shy away from trying read-across because they perceive it as too complex to get right (they might not have access to the required expertise in chemistry, toxicology, NAMs, etc.). This is where external help (consultants, consortiums, or tools like QSAR Toolbox with RAAF guidance) can lower the barrier. Ideally, as training on RAAF spreads, it becomes more routine and not a scary checklist. The article might mention that initiatives like OECD Toolbox training, ECHA’s own webinars, and documents like GRAP have made RAAF more accessible, thus mitigating the hindrance aspect. Ultimately, RAAF’s strictness protects the credibility of read-across; it hinders frivolous or poorly justified attempts (which is a good thing for the method’s reputation) while enabling solid attempts to shine through.

GRAP–Promoting Uptake through Guidance: GRAP’s intention is purely to enable–it does not impose requirements, it offers solutions and encouragement to do read-across well. It has likely had a positive effect on uptake by demystifying read-across and collating strategies. By highlighting success cases and tools, GRAP reduces the uncertainty researchers might have about “Will my read-across be accepted?” – because it outlines what acceptance criteria look like. It certainly has empowered many toxicologists in companies to give read-across a try, knowing what groundwork is needed. Additionally, GRAP emphasizes areas like *in vitro* testing that are actually more accessible nowadays (compared to running a full animal test). In that way, it channels people’s energy into creative scientific exploration (like identifying a metabolite, doing an assay) rather than defaulting to animal tests. The potential risk is that GRAP might present an ideal that is resource-intensive–for example, suggesting lots of supplemental data gathering which not everyone can do. But GRAP is not one-size-fits-all; it generally advocates proportional effort (more effort for more critical decisions). Its flexibility is an enabling factor: one can apply GRAP principles even to a simple read-across case in a lightweight way, or go all out for a complex case. The collaborative nature of GRAP (workshops, etc.) also created a community, which helps uptake because people share experiences and advice.

Do frameworks hinder innovation? A broader question: By codifying frameworks, do we risk making read-across too formulaic, possibly stifling new ideas? So far, the frameworks seem to incorporate innovation (EFSA explicitly with NAMs, RAAF via case-by-case expert judgment, GRAP by design). So they are not rigid in *how* you get evidence, just that you must have evidence. If anything, they spur innovation by identifying gaps–e.g., “*We need a way to demonstrate toxicodynamic similarity - > let’s develop a new cell assay or omics approach*.” This has been seen with the push for better *in vitro* tests for organ toxicity to support read-across.

## 7 GRAP contributions and perspective

CAAT’s Role in Shaping GRAP: Beginning with a white paper (Patlewicz et al., 2014), CAAT established a working group that developed GRAP (Ball et al., 2016; Zhu et al., 2016) and subsequently organized two workshops (Chesnut et al., 2018; Rovida et al., 2020). This body of work laid the foundation for principles now reflected in EFSA and ECHA documents. Ball et al. emphasized mechanistic and biological similarity and the explicit treatment of uncertainty—elements now embedded in EFSA’s guidance (e.g., mechanistic steps, uncertainty analysis) and required under the RAAF framework. Zhu et al.‘s focus on biological data support anticipated the integration of new approach methodologies (NAMs), now central to EFSA’s and increasingly to ECHA’s approaches. The Rovida et al. (2021) workshop, involving EFSA Scientific Committee members and ECHA experts, facilitated the transfer of GRAP’s academic concepts into regulatory practice. Notably, Susanne Hougaard Bennekou, chair of the EFSA guidance working group, co-authored the Rovida paper, underscoring the cross-pollination of ideas. Collectively, these initiatives functioned as a conceptual testing ground for approaches that later informed policy.

Advocacy for NAM/AOP Integration: The GRAP papers strongly advocated using new science to improve read-across. It provided evidence and arguments that have likely given regulators confidence to incorporate NAMs (which can be seen in EFSA’s final guidance language about NAMs lowering uncertainty, an argument the authors made in 2016 when it was less obvious). The authors would like to assert that our work on NAM-enhanced frameworks (like the flowcharts in Rovida et al.) can be used to further refine both EFSA and ECHA approaches–for instance, suggesting that a future update of RAAF or EFSA guidance explicitly include decision nodes for when to integrate NAM tests.

Driving Clarity and Consensus: The GRAP publications strongly advocated incorporating emerging scientific approaches to enhance read-across. They provided evidence and rationale that likely contributed to regulators’ confidence in integrating NAMs, as reflected in EFSA’s guidance, which recognizes NAMs as reducing uncertainty—an argument first articulated in 2016. The NAM-enhanced frameworks proposed in GRAP, including the flowcharts presented by Rovida et al., offer a basis for further refinement of EFSA and ECHA methodologies. Future updates to the RAAF or EFSA guidance could, for example, include explicit decision nodes indicating when to integrate NAM-based testing.

Continuing the Dialogue: The evolution of read-across frameworks remains an ongoing process that benefits from continued collaboration between regulatory bodies and scientific initiatives. Emerging efforts—such as potential successors to GRAP or Horizon Europe projects—can help ensure that guidance documents remain scientifically current. Rather than existing in isolation, these frameworks represent a continuum of methodological refinement driven by sustained interaction between research and regulation.

## 8 Advances in read-across methodologies and applications (2015–2025)

The decade from 2015 to 2025 has witnessed a convergence of scientific innovation, computational tools, and formal guidance that together are transforming read-across from an expert-driven art into a more standardized and defensible predictive science. NAMs–encompassing *in vitro* assays, high-throughput screening, ‘∼omics’ technologies, and AOP frameworks–have become integral to read-across workflows, augmenting traditional chemical similarity with biological context. Early efforts to incorporate biological data (Zhu et al., 2016) showed that chemicals grouped by common bioactivity profiles (e.g., similar ToxCast *in vitro* assay signatures or gene expression responses) often exhibit more reliable toxicity concordance than those grouped by structure alone. By aligning chemical categories with mechanistic pathways, researchers have enhanced the plausibility that a property observed in a source analog will manifest in the target substance. For example, multi-omics approaches combining transcriptomic and metabolomic profiling have been used to refine analogue selection and strengthen the mechanistic rationale (“mechanistic plausibility”) underpinning a read-across argument. Such strategies explicitly address one of the key historical deficiencies of read-across–the uncertainty about whether two structurally similar chemicals truly share the same toxicodynamic and toxicokinetic behavior. Indeed, a recurring theme in this period is the shift toward mechanistic similarity as a complement to structural similarity: categories are now defined not only by chemical features but also by common biological activities and AOPs. Incorporating NAM data (from bioassays, cell models, or computational biology) into read-across has been identified as one of the most important innovations to improve its acceptability. These NAM-enhanced workflows allow risk assessors to demonstrate that source and target chemicals trigger analogous key events or toxicity pathways, lending empirical support to the read-across hypothesis and thereby increasing confidence in the prediction’s scientific credibility. Another important role of NAMs is to define the boundaries of a category and to contribute to selecting the best candidate(s) as test items in new experimental methods. Practically, NAMs serve different operational roles in the two modes—pairwise concordance checks for analogue read-across versus clustering, boundary definition, and sentinel selection for category read-across—providing objective rules for domain definition and minimal testing. Parallel to these experimental advances, the period 2015–2025 has also seen rapid development of computational tools and machine learning (ML) techniques that systematize the read-across process. CEFIC’s AMBIT tool^17^, a database including more than 450.000 chemical structures and REACH dataset of 14.570 substances, supports and streamlines read-across approaches. Traditional read-across was often a manual, expert-driven selection of analogues, prone to subjective judgment. In contrast, new algorithmic approaches automate analogue identification and toxicity prediction, improving reproducibility and transparency (Luechtefeld et al., 2018). One prominent example is the Generalized Read-Across (GenRA) approach (Helamn et al., 2019), which was implemented as a workflow in the US EPA’s CompTox Chemicals Dashboard^18^ to perform automated local similarity searches and toxicity predictions. GenRA, which has still no regulatory acceptance, uses defined chemical descriptors and sometimes bioactivity fingerprints to identify suitable analogues in a consistent manner, essentially formalizing expert rules into a reproducible procedure. The OECD QSAR Toolbox^4^, a widely used platform for category formation and read-across, has been continually updated in this decade to include new profilers (for example, metabolic simulators and alerts tied to AOPs) and even an *Automated Read-Across Workflow* for certain endpoints. In 2021, the Toolbox introduced a fully automated decision-tree workflow for acute oral toxicity prediction, which applies a structured decision scheme to select analogues based on both structural and mechanistic similarity. Subsequent validation of this workflow showed it met developers’ expectations for predictive performance and provided *transparent reporting* of how predictions are derived–a critical feature for regulatory scrutiny. Beyond rule-based systems, more sophisticated ML-driven read-across models have emerged. A notable innovation is our concept of read-across structure–activity relationships (RASAR), which blends the local analogy principle of read-across with statistical learning techniques (Luechtefeld et al., 2018). RASAR models encode chemical similarity (e.g., using binary fingerprints and Jaccard distances) and then employ supervised learning to predict toxicity outcomes, effectively automating and extending the read-across logic. In a 2018 the authors reported that such RASAR models could achieve balanced accuracies on par with, or even exceeding, the reproducibility of traditional *in vivo* tests across multiple health endpoints. Likewise, *quantitative read-across* (q-RASAR) approaches have been proposed as a way to derive point estimates (e.g., predicted No-Observed-Adverse-Effect-Levels) with confidence intervals, marrying the interpretability of read-across with the quantitative rigor of QSAR (Banerjee et al., 2024). The advent of workflow tools like KNIME^19^ has further facilitated the integration of diverse data streams–chemical descriptors, bioassay results, physicochemical properties–into unified read-across pipelines. Researchers can now construct KNIME workflows that filter and weight analogues by multiple similarity criteria, apply machine learning models, and document each step, yielding a transparent *in silico* read-across process. The net effect of these computational advancements is a move toward objective, systematic, and reproducible read-across predictions, as opposed to the case-by-case expert assessments of the past (Patlewicz et al., 2019). With algorithms handling the heavy lifting of analogue selection and prediction, human experts can focus on interpretation and refinement, thereby reducing bias and improving repeatability when different practitioners perform a read-across on the same substance.

Crucially, these scientific and technical advances have been accompanied by, and indeed have driven, methodological frameworks and guidance that emphasize rigor, uncertainty analysis, and documentation. Regulatory bodies in Europe and the US recognized that increasing the success of read-across in decision-making requires not just better science, but also better structure in how read-across justifications are constructed. ECHA’s RAAF set an early precedent, which delineated criteria for evaluating read-across justifications. The RAAF formalized what regulators expect to see in a submission–for instance, clear rationale for why source and target are similar, consideration of metabolic pathways, and a robust argument addressing any differences between them. However, the RAAF was a checklist for assessors rather than a how-to for practitioners. In response, the toxicology community–via cross-sector collaborations like the t^4^ workshop and EU’s Horizon 2020 projects–developed GRAP guidance to assist in the proper construction of read-across cases. The GRAP guidance (initially articulated by Ball et al., 2016) highlighted state-of-the-art practices and common pitfalls, placing special emphasis on the consideration and expression of uncertainty, the use of biological support data, and the impact of frameworks like the RAAF on read-across strategy. A consistent message in GRAP and subsequent discussions was that read-across should be grounded in a weight-of-evidence approach, where chemistry, biology, and toxicology data are coherently combined to support the hypothesis. By 2020, an international workshop of experts (Rovida et al., 2020) reaffirmed three major technical issues that need to be addressed to improve regulatory acceptance of read-across: (i) defining similarity between source and target in scientifically meaningful terms, (ii) translating source data (especially biological activity) to predict target effects, and (iii) accounting for differences in toxicokinetics (ADME) between source and target. Each of these points maps onto the advancements of the late 2010s: the push for mechanistic similarity directly tackles (i) and (ii) by broadening similarity beyond structural features, while the integration of NAM data (including *in vitro* metabolism assays) helps address (iii) by providing evidence on comparative metabolism or kinetics. These developments were influential enough that by mid-decade regulators themselves began issuing comprehensive guidance as now EFSA in food and feed safety assessment. EFSA’s guidance explicitly encourages the incorporation of NAM data at relevant stages to strengthen the read-across case. A particular emphasis is placed on structured uncertainty analysis–assessing whether the uncertainties in the read-across prediction can be reduced to acceptable levels by using standardized methods and additional evidence (including NAM results). This mirrors the evolving consensus that transparency about uncertainty is as important as the prediction itself: a read-across argument will only be persuasive to regulators if it openly discusses its limitations and how they are mitigated. The new EFSA guidance, alongside existing ECHA and OECD frameworks, thus provides a formal scaffold that connects the scientific innovations to regulatory decision-making, ensuring that novel read-across techniques are applied in a manner that is systematic, reviewable, and fit for purpose in risk assessment.

**FIGURE 4:**
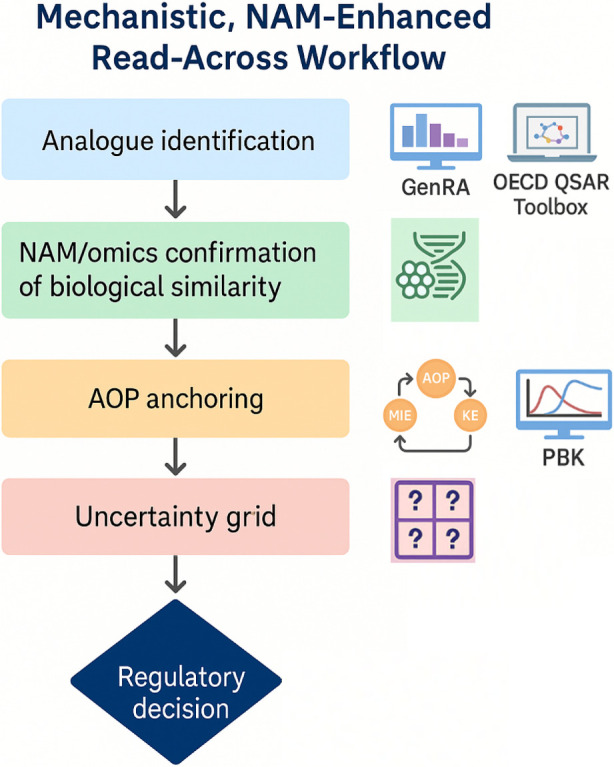
Read-across workflow based on mechanistic reasoning and NAMs.

Importantly, the synergy of these advances–mechanistic NAM data, computational workflows, and rigorous frameworks (Figure 4) – has begun to bear fruit in terms of regulatory acceptance and practical use of read-across. Regulatory agencies have shown increasing willingness to rely on read-across as part of chemical evaluations, provided the read-across is performed credibly. Under EU REACH, for example, read-across has long been allowed to fill data gaps, but many early read-across proposals were rejected due to inadequate justification of similarity or undocumented uncertainty. The innovations of the last decade directly confront those deficiencies. Where a registrant now supplements a read-across case with, say, toxicogenomic evidence demonstrating that both the source and target activate the same stress response pathway, or with a metabolic simulation showing the target yields the same reactive metabolite as the source, the read-across rationale becomes far more convincing to regulators in terms of biological plausibility. Likewise, when computational tools are used to systematically identify analogues and even quantify prediction confidence, the submission is less easily dismissed as subjective special pleading. There are signs of wider acceptance: the formation of an ICCVAM Read-Across Workgroup (RAWG)^20^ in the United States–comprising representatives from the EPA, FDA, NIEHS, DoD and other agencies–indicates broad regulatory interest in standardizing and expanding the use of read-across as part of NAMs for toxicity assessment. This workgroup explicitly seeks to catalog successful case studies and develop best practices to ensure scientific confidence in read-across, including leveraging informatics approaches to make read-across predictions more systematic and objective across regulatory contexts. International harmonization efforts via the OECD have also embraced read-across within Integrated Approaches to Testing and Assessment (IATA), often linking chemical categories to AOPs and considering read-across alongside other NAM data in weight-of-evidence evaluations. On the industry side (Tollefsen et al., 2014), practical applications of these innovations are evidenced by companies increasingly using hybrid models (combining QSAR, read-across, and *in vitro* data) to support internal decision-making and product safety assessments, thereby reducing animal testing while maintaining scientific rigor. Notably, some predictive platforms now integrate read-across with machine learning such that if a queried compound lacks data, the system can automatically find close analogues and provide a read-across prediction with an uncertainty estimate–a functionality unimaginable a decade ago. All these developments coalesce toward a common outcome: *wider regulatory acceptance, greater mechanistic defensibility, and more routine practical use of read-across in chemical safety assessment*. By anchoring read-across on firm scientific ground–using mechanistic evidence to justify why an analog is relevant and using computational methods to handle large data consistently–the approach becomes more than a contingency for missing data; it becomes a credible predictive tool in its own right. Indeed, a 2025 meta-review of 36 recent studies concluded that modern read-across methods now *“combine chemical, biological, and computational techniques”* to deliver “*quantitative, mechanistically sound”* toxicity predictions, reflecting a maturation of the field into a truly integrative science. In summary, the period 2015–2025 has solidified read-across as a cornerstone of NGRA, reinforcing it with mechanistic insight, cutting-edge analytics, and formalized best practices. These innovations directly support the overarching goals of regulatory toxicology–to arrive at reliable predictions of hazard while refining, reducing, and replacing animal testing–and they chart a path forward in which read-across is both scientifically robust and regulatorily accepted as part of the standard toxicologist’s toolkit for chemical safety evaluation.

## 9 Conclusion

Over the past decade, read-across has evolved from a predominantly structure-based inference tool into a more sophisticated, mechanistically informed approach guided by formal frameworks. ECHA’s RAAF, introduced in 2015 and expanded in 2017, provided a structured template for evaluating read-across justifications, emphasizing thorough scientific rationale and identification of uncertainties. The new 2025 EFSA guidance similarly instills a stepwise methodology–from problem formulation to uncertainty analysis–that embeds transparency and rigor into each phase of read-across, explicitly incorporating weight-of-evidence principles and NAMs to bolster confidence. Together with the community-driven GRAP recommendations, these frameworks have converged on core requirements: demonstration of sufficient structural and biological similarity between source and target, robust documentation of supporting data, and systematic accounting for uncertainty. This convergence has already improved regulatory acceptance, as agencies now routinely require that any minor structural differences be explained as toxicologically insignificant via sound scientific evidence.

Looking forward, read-across principles are poised to extend beyond conventional organic chemicals into emerging materials like nanomaterials and microplastics. Active research and regulatory initiatives are already exploring how to apply grouping and read-across approaches to these domains (Vogel et al., 2024). For example, dedicated frameworks for nanomaterials have been proposed–the EU’s recent GRACIOUS program^21^ developed a comprehensive strategy for grouping nanoforms and inferring their hazards via read-across. Similarly, risk assessors are beginning to tackle micro- and nanoplastics using read-across logic, recognizing that these particulate pollutants can be organized into categories (e.g., by polymer type, particle size, or surface chemistry) such that data from one group member inform. The underlying concepts of mechanistic similarity and trend analysis apply as much to a family of nano-scale materials or polymer fragments as they do to traditional molecules. Extending read-across to such complex materials will require new kinds of supporting data (e.g., physicochemical characterization, *in vitro* particle toxicology assays) and possibly new assessment elements to address physical hazards. However, the current activity in this field signals that the read-across paradigm is adaptable: the same principles of grouping by shared characteristics and justifying extrapolations with sound science can significantly aid the assessment of nanoparticles and microplastics, where generating complete *in vivo* datasets for each variant is impractical. Embracing these forward-looking applications, the next-generation of read-across will likely become an even more broadly applicable tool–one that not only bridges data gaps across similar chemicals, but also accelerates safety evaluation for novel materials in an era of innovation. Such expansions, undertaken carefully, will continue to fulfill the promise of read-across as a cornerstone of NGRA, enabling more efficient and humane evaluations without compromising scientific rigor.

In summary, the read-across paradigm is poised to thrive at the intersection of emerging technologies and regulatory science. It allows the introduction of NAMs not as validated alternatives to replace an animal test but as bridges to known chemicals. Breakthroughs in high-throughput ∼ omics and cell-based assays, along with computational advances like GenRA and quantitative read-across structure–activity relationships (q-RASAR), are rapidly expanding the scientific toolkit for analog identification and prediction. AOP-based workflows and multi-omics profiling now enable a deeper mechanistic contextualization of chemical similarity, linking molecular-level perturbations to adverse outcomes in a way that can greatly strengthen read-across hypotheses. GRAP is well positioned to embrace these innovations: its principles of thorough documentation, mechanistic reasoning, and weight-of-evidence flexibility provide an ideal scaffold to integrate new data streams. In essence, GRAP can act as a conceptual and practical bridge–aligning the ambitious scientific insights from NAMs, big data/AI, and AOP-informed models with the structured rigor demanded by EFSA’s and ECHA’s frameworks. By mapping novel evidence types onto established read-across criteria (e.g., using toxicogenomic fingerprints to substantiate biological similarity or using q-RASAR models to quantify predictive uncertainty), GRAP ensures that cutting-edge methods enhance, rather than undermine, the credibility of read-across assessments. This synergy promises to keep read-across practice in step with scientific progress.

Notably, EFSA and ECHA have shown increasing openness to these innovations, as reflected in guidance that explicitly allows NAM data to reduce uncertainty and justify mechanistic plausibility. Both agencies now recognize that convergence on certain key scientific principles will be essential to fully realize the potential of modern read-across. Chief among these principles are biological similarity (ensuring source and target chemicals truly share relevant modes of action or key toxicity pathways), uncertainty quantification (characterizing and, where possible, quantifying the confidence in read-across predictions) (Tate et al., 2021), and mechanistic defensibility (grounding read-across rationales in accepted mechanistic or AOP knowledge to explain why the prediction should hold true). Continued alignment on these foundational elements will harmonize the criteria by which new data and methods are judged across frameworks. In practice, this means that whether a read-across argument is reviewed under EFSA’s food safety lens or ECHA’s industrial chemicals program, it will be evaluated by similar scientific yardsticks–fostering consistency and mutual trust in outcomes.

In an aspirational view, the coming years could usher in a harmonized and scalable read-across approach that becomes a staple of regulatory toxicology across sectors. One can envision a future in which the structured workflows of EFSA and ECHA, enriched by GRAP’s best-practice guidance, are universally applied to chemicals, cosmetics, food additives, and environmental contaminants alike. In this future, risk assessors would routinely leverage *in silico* predictions, *in vitro* bioactivity signatures, and mechanistic biological data alongside traditional evidence (Caloni et al., 2022) as envisioned as NGRA (Pallocca et al., 2022), all within a unified read-across framework that transcends domain silos. Such a framework empowered by AI and machine learning (Hartung, 2016) would be both scientifically robust and highly efficient–capable of handling large chemical inventories with transparency about uncertainty and mechanistic relevance. Sure, this will require building trust into these methods (Hartung et al., 2025). This will enable both product development in Green Toxicology (Maertens et al., 2014; Maertens et al., 2024; Maertens and Hartung, 2018), aka Safe and Sustainable by Design (SSBD)^22^, and the mass-evaluation of substances needed for a Human Exposome Project (Sillé et al., 2020; 2024; Hartung, 2025; Miller, 2025; Sarigiannis et al., 2025). Ultimately, by continuing to bridge cutting-edge science with sound regulatory principles, the field is moving toward a read-across paradigm that can materially reduce animal testing and accelerate chemical safety evaluations. The alignment of GRAP’s flexible, innovation-friendly practices with the evolving EFSA and ECHA requirements sets the stage for read-across to fulfill its promise as a cornerstone of NGRA. Embracing this convergence, regulators and stakeholders can together realize a future where reliable read-across is routinely accepted across regulatory arenas–a future in which chemical hazard assessment is not only more humane and expedient, but also more mechanistically enlightened and broadly applicable.

## Footnotes

1
https://www.oecd.org/en/publications/guidance-on-grouping-of-chemicals-second-edition_9789264274679-en.html

2
https://cefic-lri.org/wp-content/uploads/2014/03/ECHA-Cefic-LRI-Read-across-Workshop-Report_171211-FINAL.pdf

3
https://www.efsa.europa.eu/en/topics/topic/uncertainty-scientific-assessments

4
https://www.oecd.org/en/data/tools/oecd-qsar-toolbox.html

5
https://www.epa.gov/comptox-tools/toxicity-forecasting-toxcast

6
https://cordis.europa.eu/project/id/681002

7
https://www.ecetoc.org/wp-content/uploads/2014/08/ECETOC-TR-116-Category-approaches-Read-across-QSAR.pdf

8
https://one.oecd.org/document/ENV/JM/MONO(2018)28/en/pdf; https://one.oecd.org/document/ENV/JM/MONO(2020)16/en/pdf

9
echa.europa.eu/documents/10162/17224/information_requirements_r6_en.pdf/77f49f81-b76d-40ab-8513-4f3a533b6ac9

10
https://www.intertek.com/blog/2025/07-23-read-across/

11
https://www.oecd.org/content/dam/oecd/en/publications/reports/2014/09/guidance-on-grouping-of-chemicals_g1ghcccc/9789264085831-en.pdf

12
https://www.oecd.org/en/publications/guidance-on-grouping-of-chemicals-second-edition_9789264274679-en.html

13
https://ntp.niehs.nih.gov/whatwestudy/niceatm/iccvam/international-partnerships/icatm

14
https://www.epa.gov/tsca-screening-tools/analog-identification-methodology-aim-tool

15
https://www.epa.gov/tsca-screening-tools/chemical-assessment-clustering-engine-chemace

16
https://19january2021snapshot.epa.gov/sites/static/files/2018-09/documents/genra_help_310818.pdf

17
https://cefic-lri.org/toolbox/ambit/

18
https://comptox.epa.gov/dashboard/

19
https://www.knime.com

20
https://epa.figshare.com/articles/poster/Interagency_Coordinating_Committee_on_the_Validation_of_Alternative_Methods_ICCVAM_Read_Across_Workgroup_Towards_Guiding_Principles_for_Read-Across_Application/13402364?file=25809635

21
https://www.h2020gracious.eu

22
https://research-and-innovation.ec.europa.eu/research-area/industrial-research-and-innovation/chemicals-and-advanced-materials/safe-and-sustainable-design_en

## References

[B1] Ball N. Cronin M. T. D. Shen J. Adenuga M. D. Blackburn K. Booth E. D. (2016). Toward good read-across practice (GRAP) guidance. ALTEX 33, 149–166. 10.14573/altex.1601251 26863606 PMC5581000

[B2] Banerjee A. Kar S. Roy K. Patlewicz G. Charest N. Benfenati E. (2024). Molecular similarity in chemical informatics and predictive toxicity modeling: from quantitative read-across (q-RA) to quantitative read-across structure-activity relationship (q-RASAR) with the application of machine learning. Crit. Rev. Toxicol. 54, 659–684. 10.1080/10408444.2024.2386260 39225123 PMC12010357

[B8] Bennekou S. H. Allende A. Bearth A. Casacuberta J. Castle L. EFSA (2025). Guidance on the use of read-across for chemical safety assessment in food and feed. EFSA J. 23, e9586. 10.2903/j.efsa.2025.9586 40726815 PMC12301701

[B3] Caloni F. De Angelis I. Hartung T. (2022). Replacement of animal testing by integrated approaches to testing and assessment (IATA): a call for *In Vivitrosi* . Archives Toxicol. 96, 1935–1950. 10.1007/s00204-022-03299-x 35503372 PMC9151502

[B4] Chesnut M. Yamada T. Adams T. Knight D. Kleinstreuer N. Kass G. (2018). Regulatory acceptance of read-across: report from an international satellite meeting at the 56^th^ annual meeting of the society of toxicology. ALTEX 35, 413–419. 10.14573/altex.1805081 30008009

[B5] ECHA (2016). “New approach methodologies in regulatory science. Proceedings of a scientific workshop,”. Helsinki. 10.2823/543644

[B6] ECHA (2017). “Read-across assessment framework (RAAF),”. Helsinki. 10.2823/619212

[B7] ECHA (2022). Advice on using read-across for UVCB substances. Helsinki: ECHA. Available online at: https://echa.europa.eu/documents/10162/11395738/advice_uvcb_read-across_en.pdf/.

[B9] EFSA Scientific Committee (2017). Scientific opinion on the guidance on the use of the weight of evidence approach in scientific assessments. EFSA J. 15, 4971. 10.2903/j.efsa.2017.4971 PMC7009893 32625632

[B10] Escher S. E. Kamp H. Bennekou S. H. Bitsch A. Fisher C. Graepel R. (2019). Towards grouping concepts based on new approach methodologies in chemical hazard assessment: the read-across approach of the EU-ToxRisk project. Arch. Toxicol. 93, 3643–3667. 10.1007/s00204-019-02591-7 31781791

[B11] Escher S. E. Aguayo-Orozco A. Benfenati E. Bitsch A. Braunbeck T. Brotzmann K. (2022). Integrate mechanistic evidence from new approach methodologies (NAMs) into a read-across assessment to characterise trends in shared mode of action. Toxicol Vitro 79, 105269. 10.1016/j.tiv.2021.105269 34757180

[B12] Hartung T. (2016). Making big sense from big data in toxicology by read-across. ALTEX 33, 83–93. 10.14573/altex.1603091 27032088

[B13] Hartung T. (2025). How AI can deliver the human exposome project. Nat. Med. 31, 1738. 10.1038/s4159102503749w 40514463

[B14] Hartung T. Whelan M. Tong W. Califf R. M. (2025). Is regulatory science ready for artificial intelligence? NPJ Digit. Med. 8, 200. 10.1038/s41746-025-01596-0 40210953 PMC11985935

[B15] Helman G. Shah I. Williams A. J. Edwards J. Dunne J. Patlewicz G. (2019). Generalized read-across (GenRA): a workflow implemented into the EPA CompTox chemicals dashboard. ALTEX 36, 462–465. 10.14573/altex.1811292 30741315 PMC6679759

[B16] Kuseva C. Schultz T. W. Yordanova D. Tankova K. Kutsarova S. Pavlov T. (2019). The implementation of RAAF in the OECD QSAR toolbox. Regul. Toxicol. Pharmacol. 105, 51–61. 10.1016/j.yrtph.2019.03.018 30970268

[B17] Leist M. Ghallab A. Graepel R. Marchan R. Hassan R. Hougaard Bennekou S. (2017). Adverse outcome pathways: opportunities, limitations and open questions. Arch. Toxicol. 31, 3477–3505. 10.1007/s00204-017-2045-3 29051992

[B18] Luechtefeld T. Marsh D. Rowlands C. Hartung T. (2018). Machine learning of toxicological big data enables read-across structure activity relationships (RASAR) outperforming animal test reproducibility. Toxicol. Sci. 165, 198–212. 10.1093/toxsci/kfy152 30007363 PMC6135638

[B19] Maertens A. Hartung T. (2018). Green toxicology – know early about and avoid toxic product liabilities. Toxicol. Sci. 161, 285–289. 10.1093/toxsci/kfx243 29267930

[B20] Maertens A. Anastas N. Spencer P. J. Stephens M. Goldberg A. Hartung T. (2014). Green toxicology. ALTEX 31, 243–249. 10.14573/altex.1406181 25061898

[B21] Maertens A. Luechtefeld T. Hartung T. (2024). Alternative methods go green! green toxicology as a sustainable approach for assessing chemical safety and designing safer chemicals. ALTEX 41, 3–19. 10.14573/altex.2312291 38194639

[B22] Miller G. W. Banbury Exposomics Consortium (2025). Integrating exposomics into biomedicine. Science 388, 356–358. 10.1126/science.adr0544 40273259 PMC12930644

[B23] Pallocca G. Moné M. J. Kamp H. Luijten M. van de Water B. Leist M. (2022). Next-generation risk assessment of chemicals – rolling out a human-centric testing strategy to drive 3R implementation. RISK-HUNT3R Project Perspective 39, 419–426. 10.14573/altex.2204051 35404467

[B24] Patlewicz G. Ball N. Becker R. A. Blackburn K. Booth E. Cronin M. (2014). Read-across approaches - misconceptions, promises and challenges ahead. ALTEX 31, 387–396. 10.14573/altex.1410071 25368965

[B25] Patlewicz G. Lizarraga L. E. Rua D. Allen D. G. Daniel A. Fitzpatrick S. C. (2019). Exploring current read-across applications and needs among selected U.S. federal agencies. Regul. Toxicol. Pharmacol. 106, 197–209. 10.1016/j.yrtph.2019.05.011 31078681 PMC6814248

[B26] Roe H. Tsai H. H. D. Ball N. Wright F. A. Chiu W. A. Rusyn I. (2025). A systematic analysis of read-across adaptations in testing proposal evaluations by the european chemicals agency. ALTEX 42, 22–38. 10.14573/altex.2408292 39584503 PMC11976166

[B27] Rovida C. Barton-Maclaren T. Benfenati E. Caloni F. Chandrasekera C. Chesne C. (2020). Internationalization of read-across as a validated new approach method (NAM) for regulatory toxicology. ALTEX 37, 579–606. 10.14573/altex.1912181 32369604 PMC9201788

[B28] Rovida C. Escher S. E. Herzler M. Bennekou S. H. Kamp H. Kroese D. N. E. (2021). NAM-Supported read-across: from case studies to regulatory guidance in safety assessment. ALTEX 38, 140–150. 10.14573/altex.2010062 33452529

[B29] Sarigiannis D. Karakitsios S. Anesti O. Stem A. Valvi D. Sumner S. C. J. (2025). Advancing translational exposomics: bridging genome, exposome and personalized medicine. Hum. Genomics 19, 48. 10.1186/s40246025007616 40307849 PMC12044731

[B30] Schneider K. Schwarz M. Burkholder I. Kopp-Schneider A. Edler L. Kinsner-Ovaskainen A. (2009). ToxRTool, a new tool to assess the reliability of toxicological data. Toxicol. Lett. 189, 138–144. 10.1016/j.toxlet.2009.05.013 19477248

[B31] Sillé F. C. M. Karakitsios S. Kleensang A. Koehler K. Maertens A. Miller G. W. (2020). The Exposome—a new approach for risk assessment. ALTEX 37, 3–23. 10.14573/altex.2001051 31960937 PMC13041581

[B32] Sillé F. C. M. Busquet F. Fitzpatrick S. Herrmann K. Leenhouts M. L. Luechtefeld T. (2024). The implementation moonshot project for alternative chemical testing (IMPACT) toward a human exposome project. ALTEX 41, 344–362. 10.14573/altex.2407081 39016082

[B33] Stanton K. Kruszewski F. H. (2016). Quantifying the benefits of using read-across and *in silico* techniques to fulfill hazard data requirements for chemical categories. Reg. Toxicol. Pharm. 81, 250–259. 10.1016/j.yrtph.2016.09.004 27612993

[B34] Tate T. Wambaugh J. Patlewicz G. Shah I. (2021). Repeat-dose toxicity prediction with generalized read-across (GenRA) using targeted transcriptomic data: a proof-of-concept case study. Comput. Toxicol. 19, 1–12. 10.1016/j.comtox.2021.100171 37309449 PMC10259651

[B35] Tollefsen K. E. Scholz S. Cronin M. T. Edwards S. W. de Knecht J. Crofton K. (2014). Applying adverse outcome pathways (AOPs) to support integrated approaches to testing and assessment (IATA). Regul. Toxicol. Pharmacol. 70, 629–640. 10.1016/j.yrtph.2014.09.009 25261300

[B36] Vogel A. Tentschert J. Pieters R. Bennet F. Dirven H. van den Berget A. (2024). Towards a risk assessment framework for micro- and nanoplastic particles for human health. Part Fibre Toxicol. 21, 48. 10.1186/s12989-024-00602-9 39614364 PMC11606215

[B37] Zhu H. Bouhifd M. Kleinstreuer N. Kroese E. D. Liu Z. Luechtefeld T. (2016). Supporting read-across using biological data. ALTEX 33, 167–182. 10.14573/altex.1601252 26863516 PMC4834201

